# Piezoelectric Materials in Civil Engineering Applications: A Review

**DOI:** 10.1021/acsomega.3c00672

**Published:** 2023-05-19

**Authors:** Abdulkadir Cüneyt Aydin, Oğuzhan Çelebi̇

**Affiliations:** Engineering Faculty, Department of Civil Engineering, Atatürk University, Erzurum 25030, Turkey

## Abstract

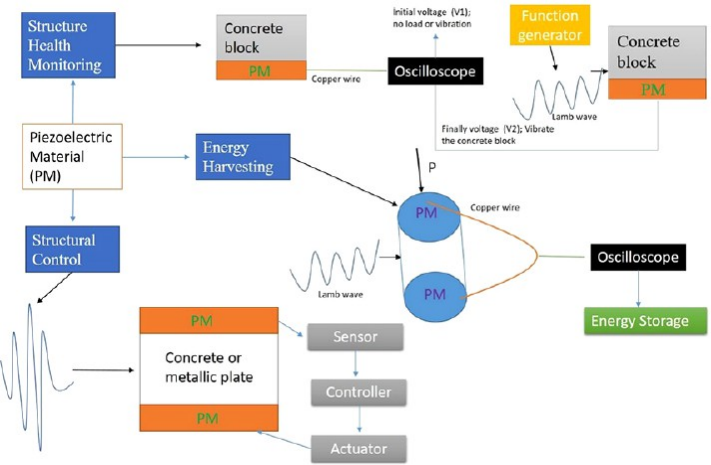

This review presents the important applications of piezoelectric materials in civil engineering in recent years. Studies on the development of smart construction structures have been carried out by using materials such as piezoelectric materials around the world. Piezoelectric materials have attracted attention in many civil engineering applications, as a result of their capability of generating electrical power when subjected to a mechanical stress, or of generating mechanical stress when subjected to an electric field. In civil engineering applications, piezoelectric materials are used in energy harvesting not only in superstructures but also in substructures, control strategies, the creation of composite materials with cement mortar, and structural health monitoring systems. With this perspective, the civil engineering applications of the piezoelectric materials were reviewed and discussed, especially for their general properties and effectiveness. At the end, suggestions were made for future studies using piezoelectric materials.

## 1 Introduction

Structures are generally built as residential, socioeconomic, and commercial concepts. Therefore, features such as life safety, comfort, and functionality are expected.^1^ In order to meet these expectations, some studies have been carried out for smart material techniques in recent years.^2,3^ As known, buildings consume 35% of the total energy worldwide.^4^ In addition, it is obvious that concrete, the main material of the building constructions, emits around 40% carbon dioxide and will create global problems in the future. In order to prevent this, it is essential for structures to produce their own energy. Structures are exposed to vibrations caused by machinery, human movements, environmental explosions, heavy vehicle passages, earthquakes, and winds.^5^ By using piezoelectric materials that create electrical energy under mechanical stress or mechanical stress under electrical energy, it will be possible to obtain energy at the same time by controlling these vibrations affecting the structure. In this way, the building will produce its own energy and reduce the energy obtained by consuming natural resources in the world.^6^ Piezoelectric materials can be used as the energy generators in buildings as an operating energy for water heating and ventilation.^7^ In addition to the energy problem, vibrations around the world from the energy released as a result of the movement of underground faults, in other words, earthquakes, cause heavy damage to the structures. In the literature, some principles have also been adopted for the protection of structures from earthquakes. These principles, based on the survival of buildings without collapse, have found their place in today’s Standards.^8,9^ Worldwide, it has been observed that after the implementation of the Standards, buildings suffered heavy damage due to earthquakes, and the loss of life decreased. However, since the Standards are based on the principle of preventing the collapse of structural members by damage or the principles of high strength, researchers have directed their studies to different concepts. Applications such as structural control and structural health monitoring have been developed in order to ensure that buildings can be immediately used before, during, and after any dynamic effect (such as earthquake and wind), as well as to ensure the high-comfort level of people.^10^ The structural control systems are examined in four groups as passive base isolation systems, active control systems, semiactive, and hybrid control systems.^11^ Although the passive base isolation systems show successful results in controlling the structures, they do not have the ability to adapt themselves against earthquakes that may occur.^12,13^ However, active control systems have the ability to adapt themselves against any earthquake by using proper system tools and control algorithms with the disadvantage of needing a large external power source.^14^ Harmonic work of the external power supply, appropriate active system tools, and control algorithms can also result in structural stability problems.^15^ Semiactive or hybrid control strategies have also been developed that use passive isolation systems to stabilize behavior by absorbing earthquake energies at the isolation level and at the same time using the self-adaptive features of active control systems against any earthquake.^16^ It is an advantage of this control system that it works like a passive isolation system in case of any power interruption in the hybrid control system, which operates with very little external power supply.^16^ The structures may lose their stability over time with the influence of dynamic loads such as wind or earthquake or static loads. The structural health monitoring strategies have been developed to detect any defects or damages that may occur within the buildings.^17^ The structural health monitoring is a kind of the damage detection performed by the dynamic characteristics of the buildings, such as natural frequencies, mode shapes, and damping, by using digital sensors.^18,19^ The researchers have developed nondestructive tests for structural health monitoring (SHM), such as ultrasonic testing (RM-Center), electrochemical impedance spectroscopy measurements (EISM) (RM-center), and pulse echo testing (RM-center).^20,21^ However, these tests have introduced researchers to a new concept of structural health studies, in terms of detecting defects in the structure globally, which is also very costly.^22^ Hence, as an environmentally friendly, light weight, and cost-effective material, piezoelectric materials turned into a tool for structural health monitoring systems-related studies.^23^ Recently, local damage determinations can be made by interacting with the structure with high-frequency excitation by using a piezoelectric transducer in the structural health monitoring technique called electromechanical impedance (EMI).^24^ Local damage determinations are made by comparing the impedance response of the damaged structure with the impedance response of the undamaged structure, by using the artificial neural network (ANN) of EMI.^25,26^ A signal analyzer or an impedance generator is used to scan for voltage on the piezoelectric material adhering to the structure. Since the electrical impedance of the piezoelectric transducer reflects the mechanical impedance of the monitored structure, damage detection can be observed.^27^ The piezoelectric materials can be used as digital sensors in damage detection by using their ability to create mechanical stress with the electric field and generate electrical energy with mechanical pressure. They can be used as an actuator to generate a control force against the forces acting within the structure.^28^ The sensitivity of the piezoelectric materials is used in damage detection and in structural control strategies by providing electrical energy for the actuator. It is also used in structural engineering to predict the early strength of a material.

The main aim of this work is to investigate the mechanical, electrical, and physical properties of piezoelectric materials, including energy harvesting, structural control, and health monitoring applications. At the end, how effective a piezoelectric material can be in civil engineering applications is discussed. What kind of innovative approach will be brought in civil engineering by using piezoelectric materials is also concluded.

The studies taken as reference in this review are given in Table 1 in general terms.

**Table 1:** Summary of Uses of Piezoelectric Materials in Civil Engineering

**Authors**	**Application**	**Method**	**Key contribution**
Chen et al. (2002)^51^	Composite beam	Dynamic stability analysis	Active control with linear quadratic regulator
Narayanan et al. (2003)^53^	Piezoelectric laminated structure	Finite element method	Sensor and actuator for active control
Schoeftner et al. (2019)^55^	One dimension rod	Single frequency harmonic test	Feed-forward technique of active control
Selim et al. (2019)^56^	GPLs reinforced composite layer	Dynamic analysis with modified Halpin–Tsai	Active vibration control for FG-V nano plates
Al-Furjan et al. (2021)^57^	GPLRC cylindrical micro shell	Semi numeric and finite element modeling	Vibration control with PD controller
Karegar et al. (2021)^58^	Reinforced concrete frame	Optimization analysis with Newmark method	Active control with GWO
Luo et al. (2022)^59^	Laminated plate	Nonlinear dynamic analysis	Active nonlinear buckling control with SMA and PZT
Chen et al. (2000)^61^	Building	Dynamic analysis	Piezoelectric friction damper (PFD)
Li et al. (2006)^62^	Beam and diagonal member	Dynamic analysis	System of isolator with Bang–Bang controller and PFD
Etadali et al. (2013)^64^	Structure	Time domain analysis	PD/PID and LQG controller for PFD
Wang et al. (2017)^67^	Stewart cubic	Newton-Euler method	6-axis orthogonal vibration isolation system
Zhao et al. (2019)^68^	Base ısolatıon system	Experimental work with real-time target machine	Active base isolation with H∞ controller
Wang et al. (2021)^69^	Piezoceramic friction damper (PCFD)	Hysteretic performance test	Takagi-Sugeno fuzzy neural network semiactive control system
Choo et al. (2022)^70^	Bridge structures	Dynamic analysis	Piezoelectric with TMD
Karayannis et al. (2015)^71^	Reinforced concrete beams	As analytical solution	Detection of flexural damage with piezoelectric sensor
Song et al. (2007)^74^	Reinforced concrete beams	High frequency excitation	Damage detection with EMI technique by using PZT sensor
Gu et al. (2005)^72^	High scaled structure	Harmonic excitation	PVDF sensor for wıreless system
Soh et al. (2000)^77^	Reinforced concrete bridges	Lamb waves	Smart piezoceramic patches in health monitoring
Alem et al. (2016)^79^	Plate-like structures	Lamb waves	Reference-free damage identification
Song et al. (2012)^83^	Concrete structures	Testing of mechanical in the concrete	Concrete piezoelectric smart material (CPSM)
Liao et al. (2011)^88^	Concrete columns	Seismic excitations	Crack damage detections with PZT sensor and actuators
Yu et al. (2013)^90^	Steel bridges	Ultrasonic nondestructive method (NDE)	Two piezoelectric sensor long-term evaluation
Hu et al. (2013)^91^	Concrete structures	Three-point bending test	Embedded piezoelectric sensor
Voutetatki et al. (2012)^92^	Concrete reinforced with FRP	Dynamic loading for numerical analysis	SHM with smart piezoelectric materials
Xu et al. (2013)^93^	concrete-filled steel tube	Wavelet packet analysis	Structural behavior
Hughi et al. (2015)^94^	RC tube and beam	Bulk wave system	Crack width monitoring system
Chalioris et al. (2015)^95^	RC beam	Typical flexural and monotonic loading	Base of PZT SHM with EMI technique
Zhang et al. (2016)^97^	Highway and airway	Diffusivity-based crack detection method	Damage detection of concrete cracks
Cahill et al. (2018)^98^	Bridge structure	Permanent magnet shaker	Energy harvesting cantilevered
Song et al. (2017)^99^	Construction infrastructure	Signal feedback from force sensor	The shape of aggregate embedded piezoelectric sensor
Zhang and Su (2017)^100^	Concrete structure, dam module	Ibrahım time domaın, shake table, compressıon test	Concrete piezoelectric smart module (CPSM) for SHM
Chen et al. (2021)^104^	Metallic structures	Lamb waves driven	Damage detection of fatigue cracks
Jiang et al. (2021)^105^	Concrete laminated interface	Push-out experimental method	Damage detection with piezoelectric smart aggregate
Pan et al. (2022)^107^	Concrete structures	Mechanical compression test	By using piezoelectric sensor and piezoelectric cement sensor with EMI for monitoring of stress and strain behavior
Kumar et al. (2013)^122^	Floor tiles	Simulation and shaker experimental method	Piezoelectric harvesting module
Lee et al. (2011)^125^	Pavement energy harvesting	Dynamic loading to pavements	Piezoelectric materials to be used in the energy conversion system on vehicle road
Wang et al. (2018)^126^	Pavement energy harvesting	Mechanical testing and simulation (MTS)	100 mm × 100 mm stacked piezoelectric energy-harvesting
Xiong et al. (2012)^129^	Pavement energy harvesting	Sinusoidal energy power output from the random external excitation	Effect of deformations of pavements on electrical energy
Wang et al. (2016)^131^	Pavement energy harvesting	Random vibratıon analysis	Response of output voltage by using 0.4 mm thickness and 30 mm diameter PZT
Yang et al. (2017)^132^	Highway traffic	Finite element method	Piezoelectric transducer
Izrin et al. (2017)^133^	Rain drop	Half wave rectifier	PVDF transducer for kınetic energy harvesting
Wang et al. (2019)^134^	Pavement energy harvesting	50 000 simulations (MTS) without cyclic loading	In order to energy harvest by using U-shaped interlayer copper foil electrode structure and lateral lead electrode structure
Tang et al. (2011)^135^	Buildings	Analytical model and experimental method	Energy harvesting from TMD using pulse width modulation (PWM) and linear quadratic Gaussian (LQG) controllers
Dutoit et al. (2005)^140^	Beam structure	Ambient vibration test	MEMS-scale device for energy harvesting
Pan et al. (2017)^141^	Cantilevered structure	Finite element analysis	Piezoelectric damper with structural vibration for energy harvesting
Xie et al. (2015)^142^	High-rise buildings	Harmonic response analysis	Piezoelectric cantilevered for energy harvesting
Priya et al. (2005)^144^	Rectangular slabs	Periodic magnetic forces	By using piezoelectric windmill and magnetic ring slabs for energy harvesting
Qian et al. (2018)^146^	Continuous bar	Spider-80X dynamic analyzer with shaker	Design of electromechanical coupling model and piezoelectric stack transducer for energy harvesting

## 2 Piezoelectric Materials

**Figure 1:**
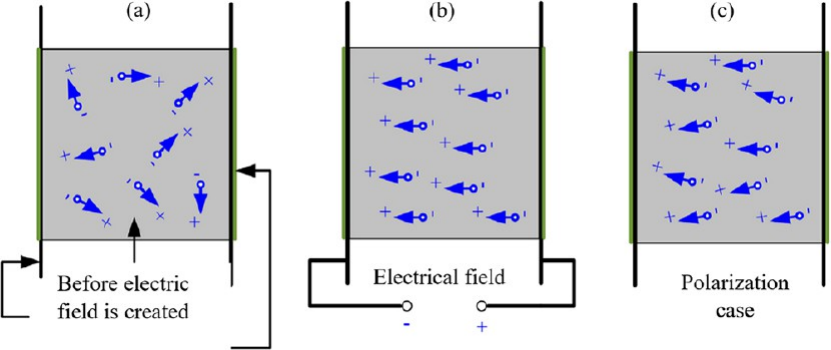
Polarization stages of piezoelectric material: (a) condition before electric field is created, (b) effect of electric field, (c) polarization condition. Reprinted with permission from ref (7). Copyright 2019 Elsevier.

**Figure 2:**
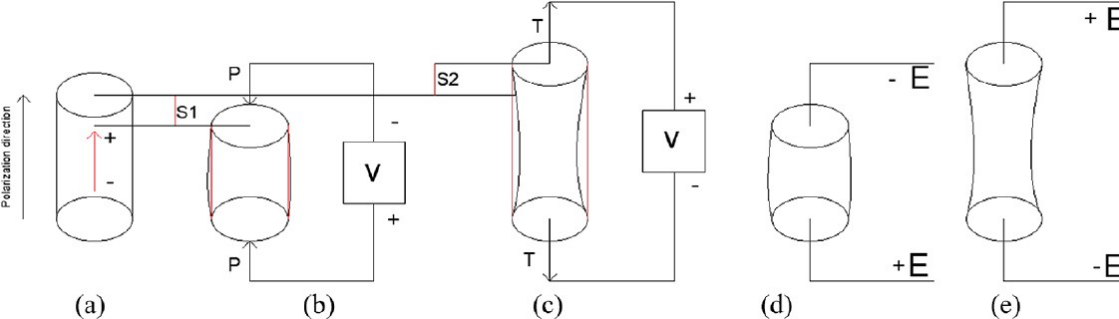
Behavior of piezoelectric materials: (a) polarization direction, (b) applying compression to the element, (c) applying tension to the element, (d) applying an electric field in the positive direction, (e) applying an electric field in the negative direction.^30^

**Figure 3:**
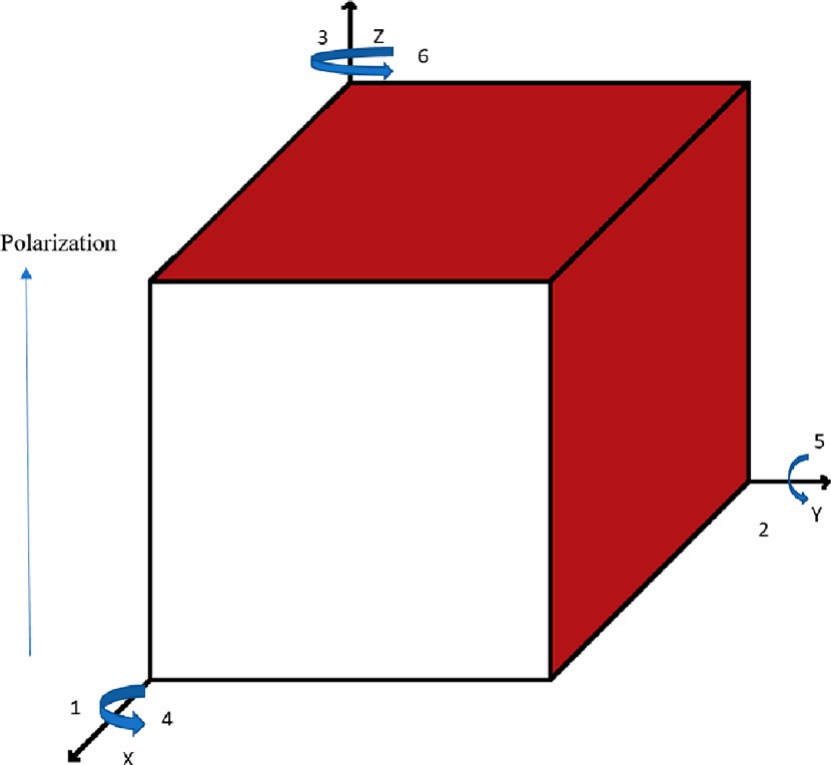
Axis sets of subscripts representing piezoelectric efficiency.

Piezoelectric materials are the materials that can detect deformation and vibration when exposed to static or dynamic loads and can work as a sensor in structural engineering and an actuator when under the influence of an electric field, also.^28,29^ In order for the piezoelectric materials to show these properties, they must be polarized as shown in Figure 1. In Figure 2, the behavior of these materials is represented. While Figure 2a shows the polarization direction, Figure 2b implies the positive voltage output as a result of applied pressure force, and Figure 2c presents the negative voltage output for a reversed force. The elongation of the piezoelectric material is determined in Figure 2d, in the polarization direction, where the shortening is presented in Figure 2e, reverse for the applied voltage. Figure 2b and c represents the generator producing electric energy in response to mechanical motion, where Figure 2d and e imply the motor behavior of the piezoelectric material showing the generation of electrical energy as a result of mechanical motion.^30^ The efficiency of the piezoelectric materials is used in eq 1 for the production of electric energy as a direct effect and eq 2 for the generation of mechanical stress as a reverse effect.^31,32^ In Figure 2b, electrical energy is produced by throttling as much as S1 with the pressure stress *P* acting on the piezoelectric material. In Figure 2c, the piezoelectric material exposed to the tensile stress (*T*) can stretch up to S2 and generate electrical energy. In addition, in Figure 2c and d, mechanical stress is produced by deforming the electric fields by *S* in the + and – directions, respectively, when applied to the piezoelectric material.
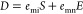
1
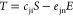
2Where *T* is the stress, *D* electrical displacement, *S* strain, *E* electric field, *c*_ji_ anisotropic elastic stiffness coefficient, *e*_jn_ and *e*_mi_ piezoelectric stress coefficients, and *e*_mn_ represents dielectric conductivities for constant stress. In addition to these parameters, the electromechanical coupling coefficient *k*, piezoelectric voltage coefficient *g*, and piezoelectric charge coefficient *d* are very important for the piezoelectric efficiency.^30,33^ The subscripts specified in the equations are related to the axes defined in Figure 3. For example, it is expressed as the unit electric displacement applied in the third direction, the stress it creates in the third direction, or the electric field created by the unit stress applied.^34^

### 2.1 Crystals

The piezoelectric materials with crystalline structure are examined in three groups: quartz (SiO_2_), tourmaline, and Rochelle’s salt. Quartz has striking properties, like high stiffness, safe, longevity, high-temperature resistivity, and durability.^30,35^ Since it is not likely to work in high-frequency excitation, it is not considered very suitable for use in damage detection and structural control applications. However, the mechanical strength and low CO_2_ emission conveyed the quartz as a source of aggregate for concrete industry to form an integrity with cement-based materials.^36^ Tourmaline and crystalline tourmaline have a high piezoelectric voltage coefficient (g) and are used in hydrophones, pressure measuring devices, to control the frequency in the propagation of radio waves.^37^ Rochelle’s salt, also known as potassium sodium tartrate, is a synthetically produced piezoelectric material with high chemical sensitivity. Furthermore, the Rochelle’s salt is not affected from the adverse environmental conditions and has a very high piezoelectric constant.^35^

### 2.2 Piezoceramic Materials

The most well-known piezoceramic materials are the lead zirconate titanate (Pb–Zr–Ti) and the barium titanate (BaTiO_3_).^37^ These materials are mostly preferred in actuator and transducer applications. It is applied by producing desired discs, cylinders, plates, or thin films from ceramic materials in powder form.^38^ Generally, they are divided into two categories, hard materials and soft materials.^39^ Hard type materials can withstand high electrical impulses and mechanical stresses; therefore, they are suitable for high voltage and high-power generator and transducer applications. Soft type materials have high sensitivity and dielectric constant. However, since internal heating may occur in these materials, they are unsuitable for use under heavy conditions. They are suitable for use in a variety of sensors, low-power motor-type converters, and low-power generators.^39^ The PZT, consisting of lead, zirconate, and titanate (Pb–Zr–Ti) elements, is the most commercially common electronic ceramic, which is proper for various additives and striking with its superior properties. Compared to barium titanate (BaTiO_3_), the PZT materials have higher sensitivity and higher operating temperature capability. The PZT is physically strong, chemically inert, and relatively inexpensive to manufacture. However, it can cause negative effects on the environment in terms of containing lead. The PZT dielectric constant and curing temperature (working temperature) are relatively high. The PZT-based energy harvesters are more efficient at converting mechanical energy to electrical energy, due to their high electromechanical coupling coefficients.^40^

Barium titanate is also used for the production of electronic elements such as capacitors, actuators, sensors, and transducers.^34^ It is an insulator in the pure form and becomes a semiconductor by adding a small amount of metal to it.^34^ Lead-free barium titanate is one of the alternatives for PZT, in terms of not making any negative contribution to the environment.^41^ Attention should be paid to the PZT’s Young’s modulus for durability, high piezoelectric voltage coefficient for electrical efficiency, and high strain coefficients, which are used in civil engineering structural health monitoring areas. In the literature that provides this efficiency, PZT with the code PIC 255 has a thickness of 0.5 mm and is produced in the desired format. The tensile constant of this material is −7.15 Cm^–2^ and the Young’s Modulus is 62.89 GPa. The reason why PZT is used as a very thin element is due to its high capacitance for electrical energy generation.^42^

### 2.3 Polymers

The most well-known polymer for its piezoelectric properties is polyvinylidine di fluoride (PVDF).^43^ As a polymer, PVDF is flexible and resistant to mechanical destructive forces.^44^ Since the production of ceramic materials is more difficult and fragile than that of polymers, the importance of polymers has increased. Polymers are more suitable for applications with large and complex shapes.^40^ It can be injectable, molded, or welded and is widely used in lithium-ion batteries as well as in the chemical, semiconductor, medical, and defense industries. It is also preferred as cross-linked closed-cell foam, which is increasingly used in aerospace applications.^45^ The PVDF is nontoxic and is widely used as insulation in electrical cables, due to its combination of flexibility, high-temperature and chemical corrosion resistivity, low thermal conductivity, and lightweight. The piezoelectric properties of PVDF are utilized in the manufacture of tactile sensor arrays. The piezoelectric properties of PVDF are used for the manufacture of tactile sensor arrays; inexpensive strain gauges, and lightweight sound transducers.^44^ Apart from PVDF, piezoelectric polymers include Parylene C, cyclo-olefin polymers (COP), microfibrillated cellulose (MFC), and polypropylene (PEP). Parylene C is generally preferred in MEMS microphones, cyclo-olefin polymers are preferred in loudspeakers, and MFCs are preferred in acoustic emission sensors. PVDF, polypropylene, and fluorinated ethylene propylene are generally used in accelerometer sensor productions. However, PVDF has advantages over other piezoelectric polymers used in the production of accelerometers in that it is more sensitive, operates in the high frequency range, and measures acceleration in larger ranges. In this case, when considered from the civil engineering perspective, it is foreseen that PVDF should be preferred in order to record acceleration measurements more precisely in high vibration ranges.^46^ Piezoelectric PVDF foils look very promising compared to similar PZT ceramic solutions.

There are a variety of the most widely used piezoelectric materials, which have some advantages and disadvantages over each other. The advantages of PVDF over PZT can be listed as follows: PVDF is cheaper than the PZT, does not age, and does not break under harsh conditions.^41^ Since the PVDFs can be produced as fibrous, they form a better composite with other building materials. PVDF is noncombustible, very resistant to chemicals, and can be processed by injection molding. Since the polymer piezoelectric materials are mechanically flexible compared to the ceramics, they have a longer life in energy harvesting and are more resistant to high vibrations than ceramics. The polymer piezoelectric materials can be produced more easily and require complex shapes. PVDF produces a higher voltage and electric field in response to mechanical stress in sensor application. Compared to the piezoceramics, the *g* constant of PVDF, i.e., voltage coefficient, is greater.^47^ PVDF is more commonly used in sensor applications. Compared to PVDF ceramic piezoelectric materials, it is easy to manufacture, convenient, flexible, robust, and light. The advantages of PZT over PVDF can be listed as follows: PVDF’s relatively low curing temperature (105 °C) and material stretching and polarization difficulty. PZTs are used as standalone sensors and actuators, while PVDFs are used as sensors or actuators. PVDF has lower piezoelectric coefficients than PZT.^44^

In civil engineering applications, in order to obtain energy from PVDF under any vibration effect, the thickness of the PVDF must be at the micrometer level. In addition, piezoelectric constants *d*_13_ and *d*_33_ of PVDF should also be preferred at high values. Song et al. (2017) used 50 μm thick PVDF films with a Young’s modulus of 4.18 GPa and d_13_ and d_33_ coefficients of 23.4 and −32.5 Pc/N, respectively, to obtain energy from vibrations.^48^

The piezoelectric coefficients of the most widely used piezoelectric materials today are given in Table 2 and their superiority over each other is given in Table 3.

**Table 2:** Piezoelectric Coefficients of Piezoelectric Materials^34^

Materials	Typical	*d* (Pc/N)	*g* (× .10^–3^ V m/N)
Quartz	Cyrstal	2.3	50
Rochelle salt	Cyrstal	2300	Very low
Barium titanate	Ceramic	191	12.6
PZT	Ceramic	289	25.9
PVDF	Polymer	–33	–339

**Table 3:** Advantages and Disadvantages of Piezoelectric Materials Relative to Each Other

Parameter	Barium titanate^34,41^	Lead zirconate titanate^37−40^	Quartz^30,35^	PVDF^40,41,43−47^	Rochelle salt^35^
Range of frequency	0–70	0–100	very low	0–10	very low
Curing temperature	120	200	very high	105	225
Environmental impact	affected	affected	not affected	affected	not affected
Negative impact on environment	no	yes	no	no	no
Mechanical property	rigid	rigid	rigid	flexible	rigid
Usage area	sensor/actuator				
Energy production	high	very high	very low	normal	normal
Density (g/cm^3^)	6.02	6.53	2.65	1.78	1.79

## 3 Civil Engineering Applications of Piezoelectric Materials

The main applications of piezoelectric materials in civil engineering are energy harvesting systems, structural control, structural health monitoring, mechanical observations, deformation measurements, and seismic base isolation systems. In this work, the piezoelectric materials were examined under three headings: structural control systems, structural health monitoring, and energy harvesting systems.

### 3.1 Structural Control Applications

One of the main applications of piezoelectric materials is their use as actuators. These actuators are also widely used in structural control systems. These types of applications are presented in this section.

Piezoelectric materials have been applied in structural control systems as actuators due to their fast response, high-output force, small size, and light weight.^49,50^ Piezoelectric actuators are generally placed between the movable base and the upper platform, producing a certain control force while transferring the vibrational energy from the base to the upper platform. Active vibration control is carried out with control forces by actuators using controllers, such as a linear quadratic regulator (LQR).^51^ In the literature, the control of displacements and accelerations in structural members obtained by using piezoelectric materials has received great attention recently.^52,53^ Zarei et al. (2018) investigated the dynamic behavior of a beam wrapped with a piezoelectric material used as a sensor and actuator with the help of the differential quadratic method.^54^

**Figure 4:**
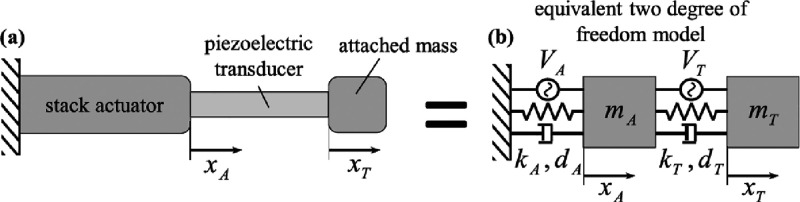
Suggested dynamic model to control stress: (a) the geometry of the system, (b) the dynamic model of the system. Reprinted with permission from ref (55). Copyright 2019 Wiley.

Schoeftner et al. (2019) actuated a piezoelectric transducer to reduce the dominant stress to zero in a one-dimensional bar, by using the feed-forward technique.^55^ They transformed the two-degree freedom equations of motion and created into differential equations related to displacement, voltage, and electric field. They developed a static- and frequency-dependent mathematical model to activate the piezoelectric transducer. To verify the simulation results based on the mathematical model, they experimentally performed a single-frequency harmonic excitation test. The mathematical model in the study is shown in Figure 4. In Figure 4a, the stacked actuator is connected to the transducer with a single bonded mass at the free end of the piezoelectric transducer. Here, piezoelectric elements are characterized by mass, stiffness, and damping. Figure 4a is decomposed as a system with 2 degrees of freedom, and a dynamic model as in Figure 4b is obtained.

Here *m*_A_ and *m*_T_ are the masses of the stack actuator and the piezoelectric transducer, respectively. Since the tension was assumed as uniformly distributed across all sections, the axial stress can be directly changed by the piezoelectric transducer force *F*_T_ and the stack actuator force F_A_. Therefore, in the presence of an electric field in the stack actuator, the *F*_T_ force can be reset by an electric field applied to the transducer. Within this perspective, eqs 3 and 4 are evaluated, by applying Newton’s dynamical law in the Laplace field.
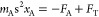
3
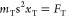
4Here, *s* is the coordinate of the Laplace transform, and *x*_T_ and *x*_A_ are the absolute Laplace-transformed displacements of the two effective masses. It has been transformed into two linear equations relating displacements and the electric field so that the internal forces specified in the equations can be represented. The viscoelastic properties of piezoelectric materials are taken into account to account for slight damping. Considering these features, the structural behavior is specified in eqs 5 and 6.
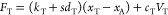
5
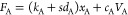
6Here, the elastic stiffness of the member is defined as *k*, and the viscous parameter *d*. The voltage applied to the element is represented by *V* and the piezoelectric constant *c*. It is the basic logic to calculate the displacement and internal force against the voltage given to the element in the equations. Further processing follows by assuming that one of the two electrical voltages in the two piezoelectric elements disappears and calculating the corresponding transfer functions.

**Figure 5:**
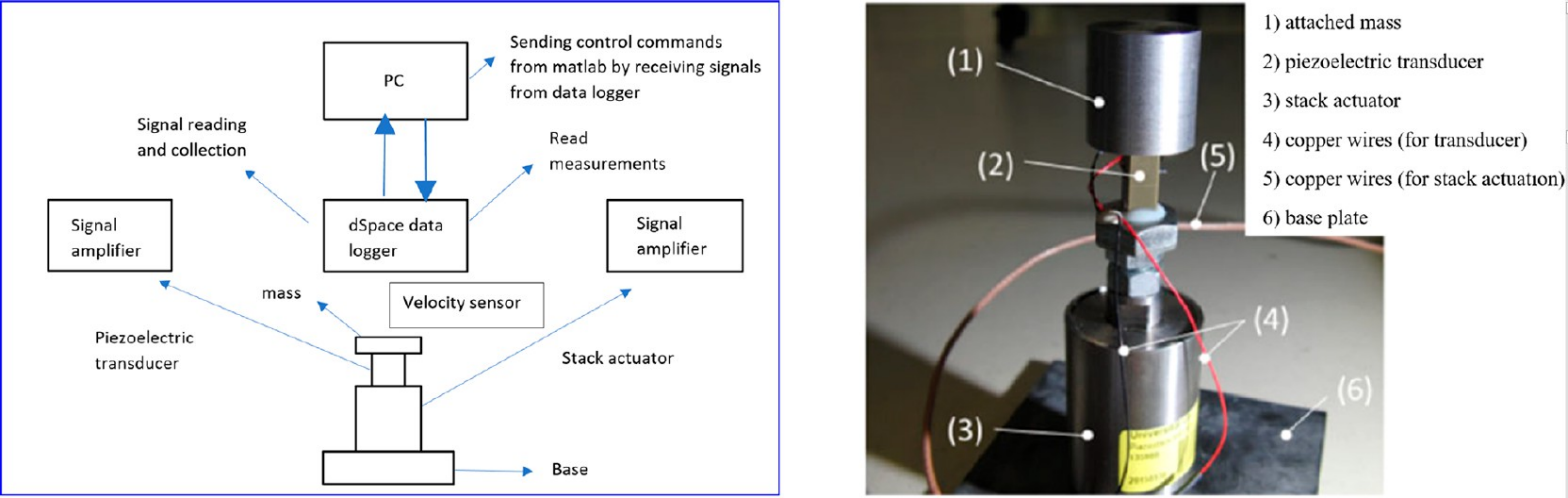
Experimental setup for the single frequency harmonic test. Reprinted with permission from ref (55). Copyright 2019 Wiley.

In order to verify the numerical theory, the experimental setup shown in Figure 5 was prepared, and the single-frequency harmonic test was performed in five steps. First, FRF measurements were made before the 30 min test. Then, a 30 min force-controlled test was performed. After 30 min of testing, FRF measurements were made again. After the destructive test, the voltage of the piezoelectric transducer was set to 0 and FRF measurements were made again. Numerical and experimental results have shown that strain control is achieved with visible damage to the piezoelectric transducer resulting in a significant change in the first eigen frequency.

**Figure 6:**
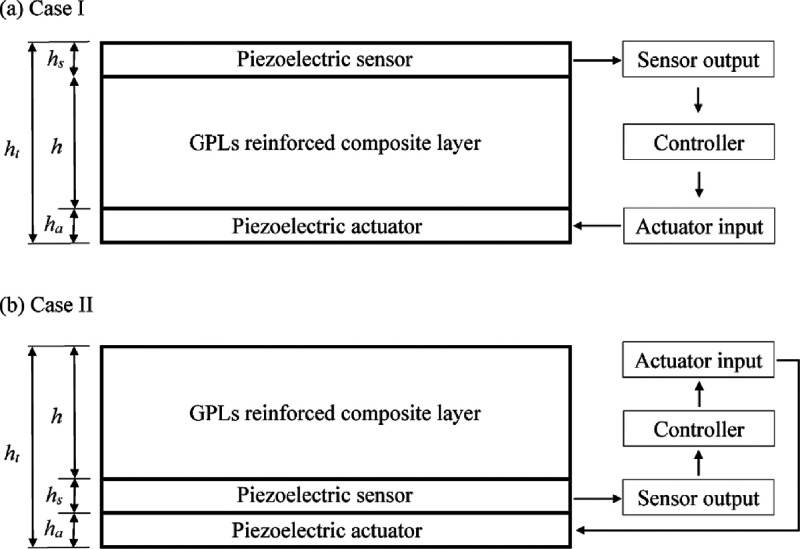
Control process considering the two positions of the piezoelectric sensor and actuator layers. Reprinted with permission from ref (56). Copyright 2019 Elsevier.

Selim et al. (2019) performed active vibration controls of functionally graded graphene nanoplatelets. These were integrated with piezoelectric layers of composite plates.^56^ The volume fractions of piezoelectric layered GPL (graphene nanoplatelets), distribution patterns of GPLs, ratio of the total thickness of the plate to the width ratio, ratio of the piezoelectric layer thickness to the total plate thickness, and effects of boundary conditions are provided. The effects of such parameters on the natural frequency increase between open and circuit conditions are also discussed. On the other hand, a constant speed feedback controller is used for active vibration control of GPL reinforced composite plates integrated into piezoelectric layers. The two positions of the piezoelectric sensor and actuator layers are called as the sensor and actuator layers. The piezoelectric sensor is considered to be on either the opposite or the same side of the plates (Figure 6).

In the study, a piezoelectric integrated layered composite plate with 4 different graphene placements was considered. In the UD model, the graphene volume ratio is uniformly distributed throughout the thickness. In the FG-O model, the graphene content is placed more in the middle and less toward the top and bottom. In contrast, less graphene content was placed in the middle of the GPL-reinforced composite part with the FG-X distribution, and more graphene content was placed both above and below it. Additionally, in the FG-V model, there is more graphene content at the top and less graphene content at the bottom. At the end of the study, it has been shown that placing the sensor and actuator on the same side as a result of increasing the thickness of the piezoelectric layers gives successful results in active vibration control for the FG-V model.

**Figure 7:**
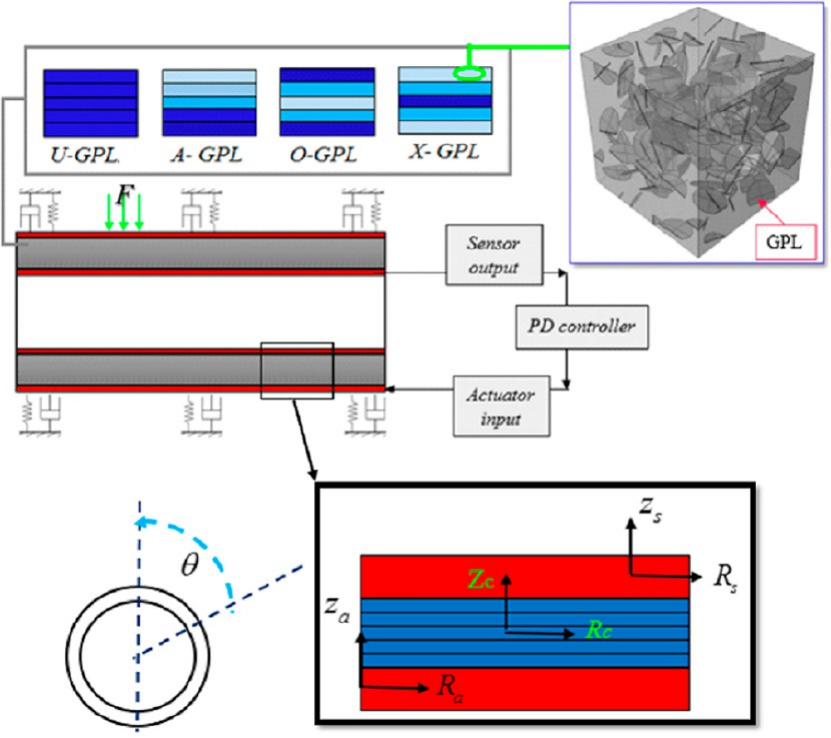
Diagram of an intelligent GPLRC cylindrical microshell under an external load and surrounded by a viscoelastic foundation. Reprinted with permission from ref (57). Copyright 2021 Elsevier.

A quasi-numerical and finite-element modeling for the vibration control of a smart shell powered one was established by Al-Furjan et al.^57^ using graphene nanoplatelets under external load. The quasi-numerical method was developed by using the first-order shear deformation theory (FSDT), based on strain–stress relations. The mixture rule was used for the bulk density and Poisson’s ratio, where the Halpin–Tsai theory was used for the modulus of elasticity. A proportional derivative (PD) controller is used to control the sensor output while external voltage is applied to the sensor layer. Boundary conditions are derived through the governing equations of the GPLRC (graphene nanoplatelets composite) cylindrical shell surrounded by PLSA (piezoelectric layer sensor actuator) using an energy method, known as Hamilton’s principle. Finally, the evaluated equations were solved by using a generalized differential quadrature method (GDQM). A finite-element model was created to simulate the response of the GPLRC. An intelligent GPLRC shell integrated with piezoelectric layers and subjected to an external load is shown in Figure 7. A PD controller is used for intelligent control of the vibrating structure, and a viscoelastic foundation is used to make the existing structure more robust.

**Figure 8:**
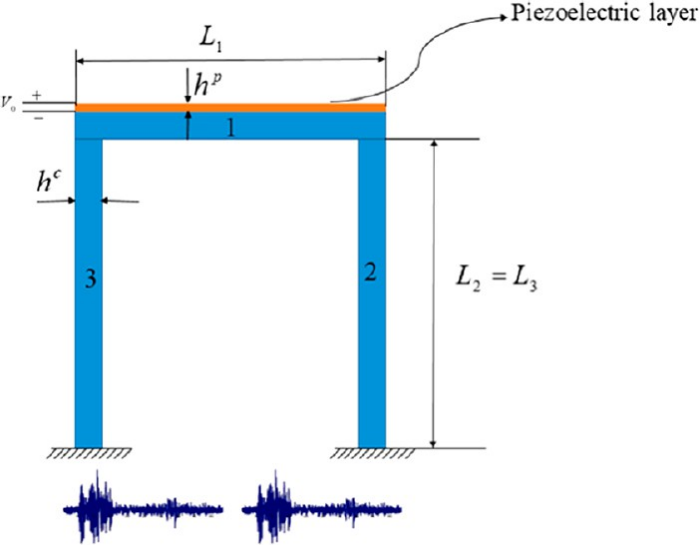
Concrete frame reinforced with smart layer under earthquake load. Reprinted with permission from ref (58). Copyright 2021 Elsevier.

Karegar et al. (2021) performed the intelligent control and seismic analysis of piezoelectric layered concrete frames based on mathematical modeling.^58^ The main purpose of the study is to investigate the effects of external stress, smart layer thickness, and boundary conditions on parameters such as structural damping and displacement of the frame. The optimization of the framework is based on the Gray Wolf algorithm (GWO), fits the gray wolves’ leadership hierarchy and hunting mechanism. The mathematical theory of hyperbolic shear deformation (HSDT) was used to model the planar frame. The governing equations are derived using Hamilton’s principle. In addition, the differential quadratic (DQ) and Newmark numerical methods were used to investigate the earthquake response of a concrete frame. Figure 8 designed to observe the earthquake response of a concrete frame with a smart layer.

**Figure 9:**
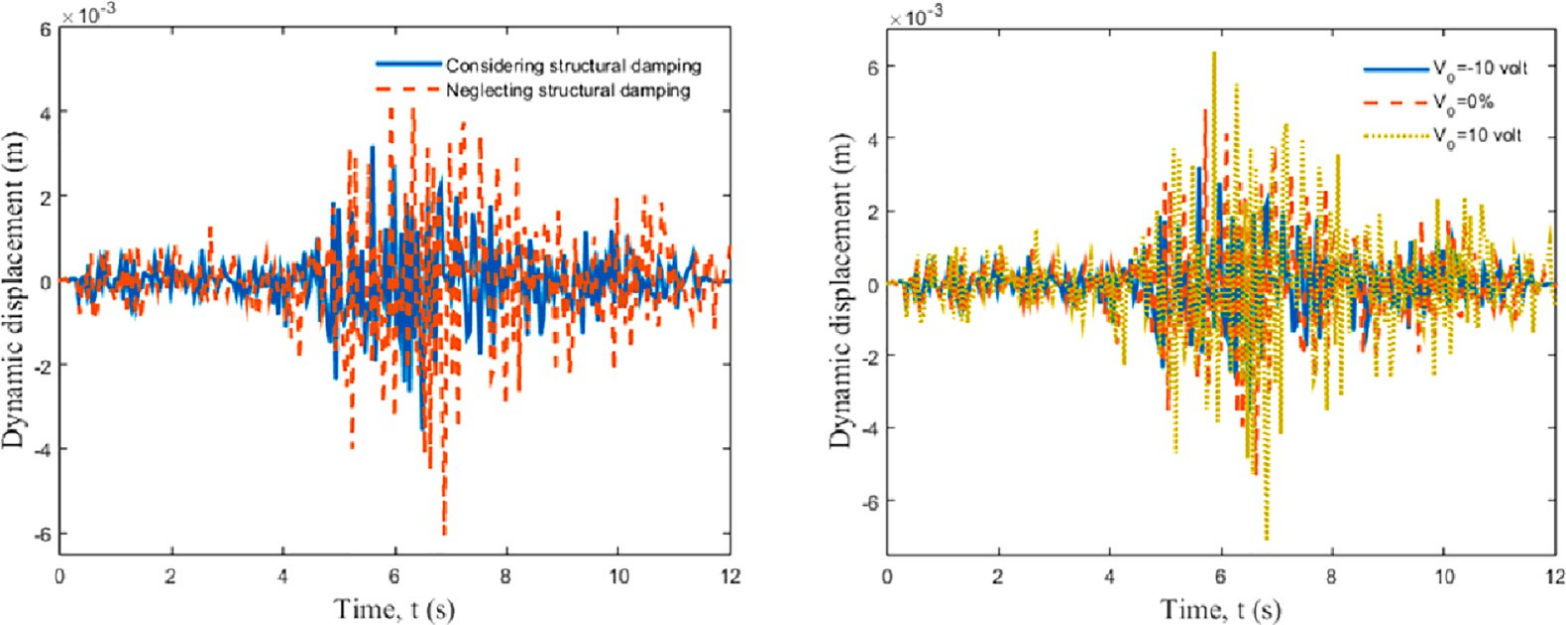
Dynamic displacements as a result of taking into account the damping and external voltage effect. Reprinted with permission from ref (58). Copyright 2021 Elsevier.

The study results showed that applying a negative external voltage reduces the maximum dynamic displacement of the frame. It was also determined that the displacement of the frame decreased when the structural damping was taken into account, and the displacement decreased as the thickness of the smart layer increased (Figure 9).

Luo et al. (2022) conducted an experimental study on active nonlinear buckling control of optimally designed laminated plates with a new control method using shape memory alloy (SMA) and piezoelectric (PZT) actuators.^59^ In this control method, SMA actuators are used to control the magnitude of plate buckling deformation, while PZT actuators are mainly used to control the direction of plate buckling deformation by inducing the initial buckling in a desired direction.

Members such as beams and plates are generally used in active vibration controls by using piezoelectric materials. For the active vibration control, structural dynamic analyses were carried out by using the finite-element model for the proposed system. An external voltage is applied to control the possible negative conditions, such as displacement and stress, caused by the dynamic vibrations of piezoelectric actuators. It has been observed that the control forces against vibrations are produced by piezoelectric actuators, where this response is a result of the externally supplied voltage. It has also been determined that control algorithms such as PD (proportional derivative), PID (proportional integral derivative), DQM (differential quadratic method), GWO (Gray Wolf algorithm), and LQR (linear quadratic regulatör), which are compatible with the active vibration control system, are used to generate these control forces. The control forces vary depending not only on the control algorithm, thickness, width, and length of the piezoelectric material but also the voltage magnitude from the external environment. In this section, it has been shown that graphene nanoplates are widely used together with piezoelectric materials for the active control of composite sheets or beams. It is thought that graphene, which has superior mechanical properties, increases the electrical properties of polymer piezoelectric materials,^60^ enabling them to produce control forces quickly under the influence of an applied voltage. However, studies have shown that the placement, thickness, and aspect ratio of graphene relative to the piezoelectric layer are also important. It has been understood that active vibration controls are performed on noncomplex elements such as beams and plates. These studies were generally carried out under the influence of low frequency excitations. In the future, it is thought that active vibration control studies can be carried out for the structural members under the influence of vibrations. In fact, it has been predicted that active vibration control of multilayered structures can be made with actuators made of graphene, which increases the electrical properties of piezoelectric materials. It is expected that the piezoelectric material to be used in active vibration controls will have both the flexibility and strength to meet the vibrations and strong electrical properties for the power supply. Considering that PVDF is flexible but its electrical properties are bad, it is expected that graphene acting together with PVDF will give good results.

**Figure 10:**
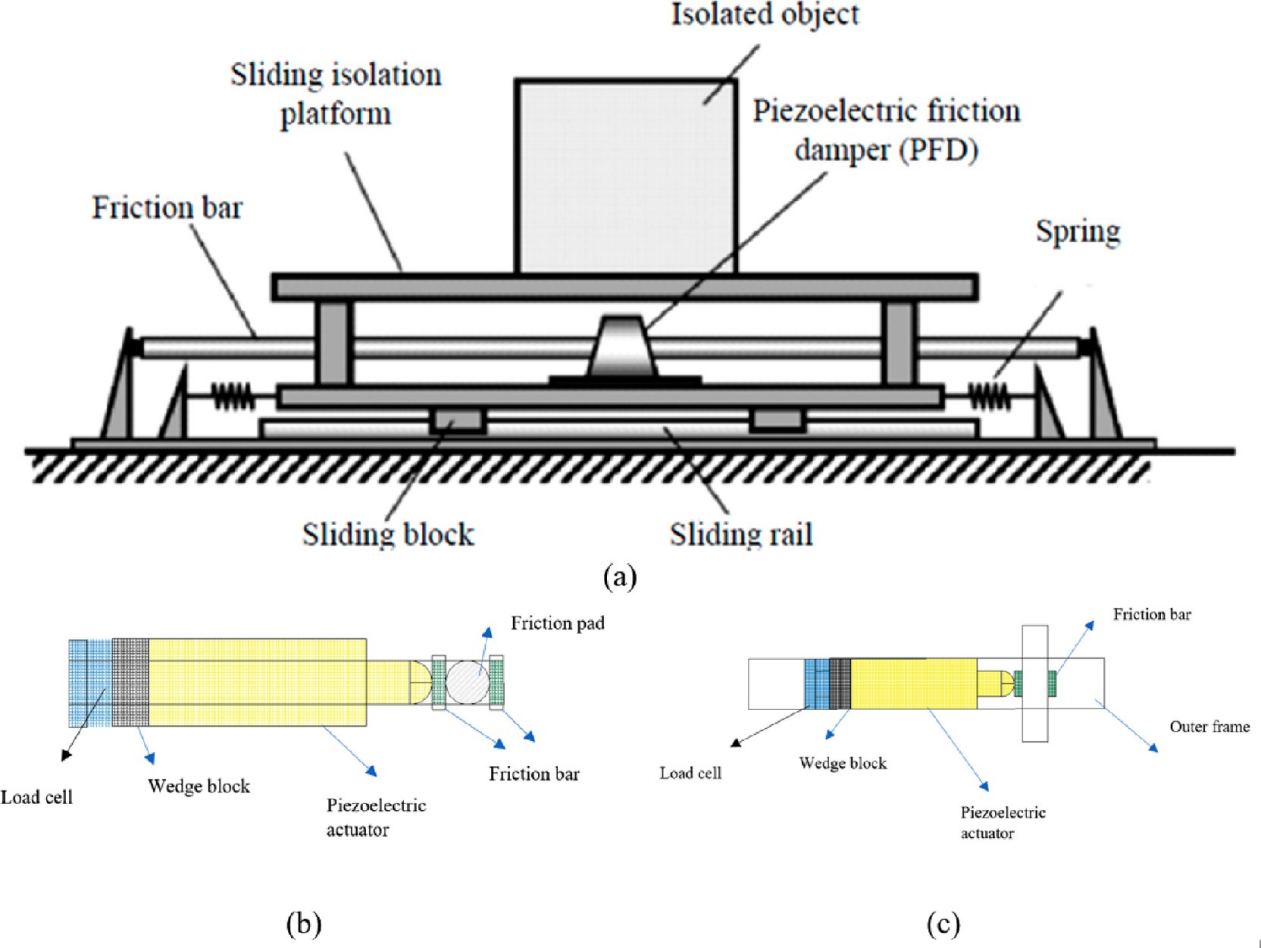
Piezoelectric smart ısolatıon system: (a) the parts that make up the isolation system, (b) the schematic of piezoelectric friction damper; (c) connecting the piezoelectric actuator to the frame. Reprinted with permission from ref (65). Copyright 2010 Elsevier.

Besides the active vibration and displacement controls, piezoelectric materials have also been used as a damper by placing the piezoelectric materials between the building support bracket and the ground.^61^ Structural response (acceleration, translation, etc.) can be reduced with piezoelectric materials integrated into the friction damper for a contact force adjustment. Li et al. (2006) proposed a piezoelectric friction damper. The proposed friction-based dampers are connected to the ground floor beams and cross members of the structure. They used a bang–bang controller to adjust the friction force.^62^ The simulation results applied to a 3-story building under the influence of El Centro earthquake data showed that the floor accelerations and translations decreased significantly. In a similar study, Chen et al. (2004) performed a numerical simulation, a semiactive control algorithm, which is to adjust the piezoelectric friction damper and contact forces, for a 20-story steel structure.^63^ Etadali et al. (2013) applied structural control by isolating a structure with piezoelectric friction dampers.^64^ In this study, seismic control of a comparatively isolated building equipped with a piezoelectric friction damper (PFD) is developed by using PD/PID and linear quadratic Gaussian (LQG) controllers. It was optimized to create a balance between the performance and robustness of the system using the genetic algorithm technique so that the control forces can be easily estimated. A piezoelectric smart tracking system (PSIS) consisting of a set of guide rails, slide blocks, linear springs, and piezoelectric friction dampers has been developed (Figure 10a).^65^ When the PSIS is subjected to an earthquake excitation, the relative motion between the friction bar and the friction pad generates a shear force. The resulting slip force can be controlled by a piezoelectric actuator. The piezoelectric actuator is embedded in the friction damper, as shown in Figure 10b to generate a controllable force vertically. The vertical (contact) force in the PFD can be controlled by varying the input voltage of the piezoelectric actuator. A pair of wedge blocks is used to pretighten the piezoelectric actuator. Also, a load cell was placed in the PFD to measure the vertical force (Figure 10c). Lu et al. (2009) used the system shown in Figure 10 to protect structures from near-field earthquakes.^66^

**Figure 11:**
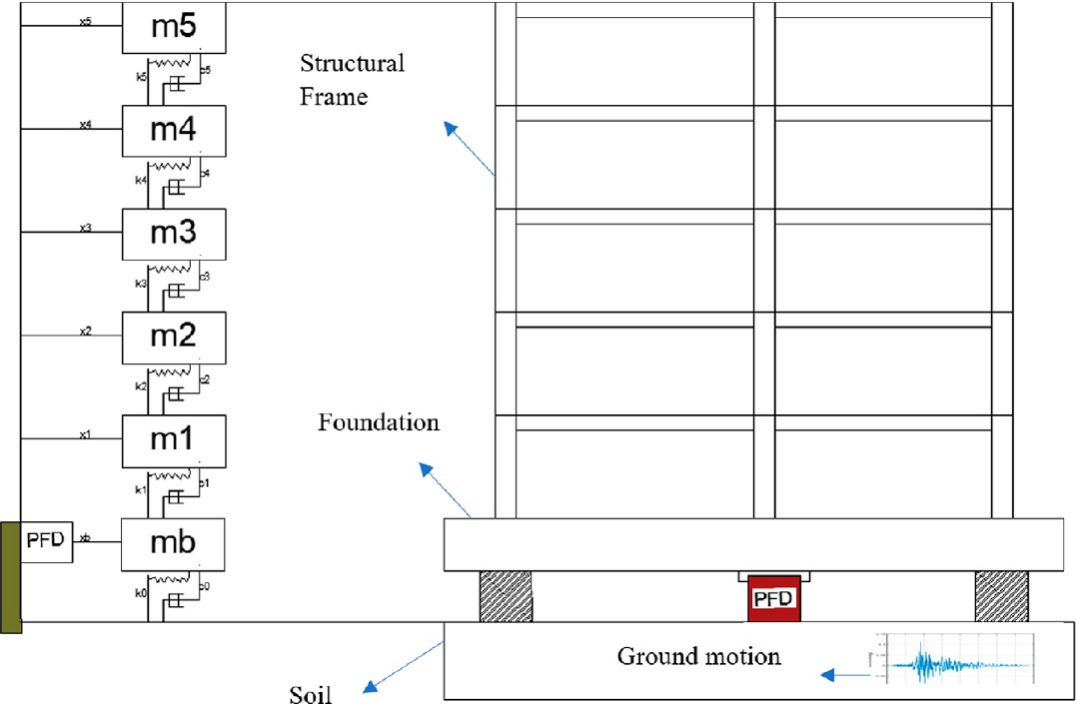
(a) Adaptation of the insulation system to the building. (b) Simulation results.

Seven earthquake records were used to investigate the performance of the proposed controllers in the time domain. It has been determined that the proposed control system leads to a decrease in base displacements and floor accelerations for the earthquakes far and near field. The model is presented at Figure 11.

**Figure 12:**
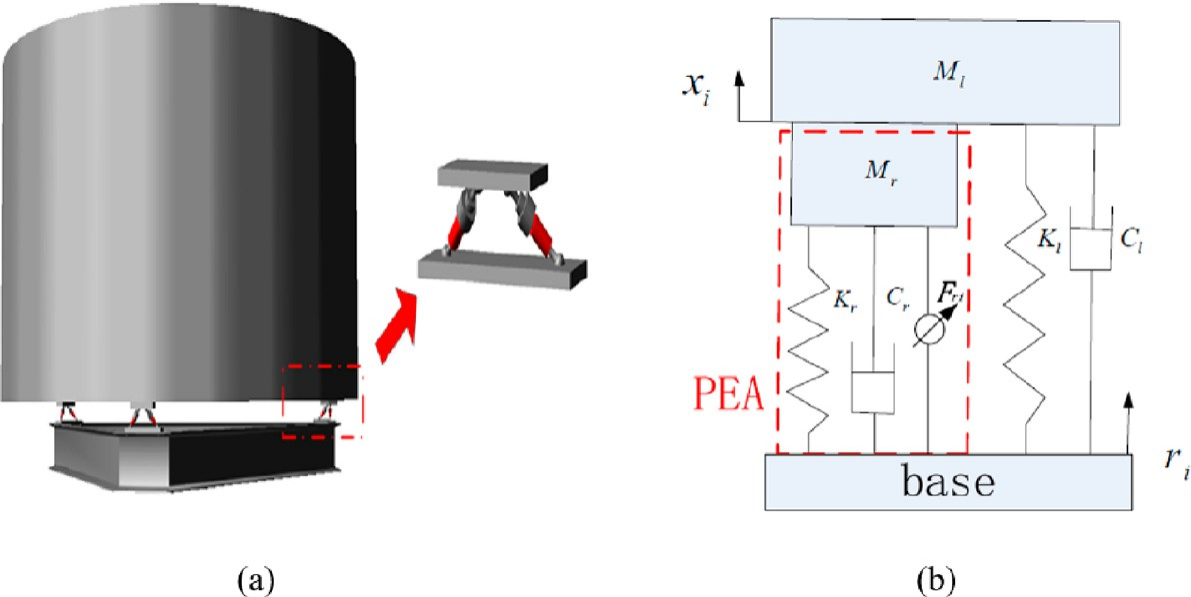
6-axis orthogonal vibration isolation system: (a) vibration isolation system, (b) the dynamic model of ısolation system. Reprinted with permission from ref (67). Copyright 2017 JVE International.

Wang et al. (2017) designed a 6-axis orthogonal vibration isolation platform based on PEAs (piezoelectric actuators) that meet the demands of heavy load, small installation space, and multifreedom vibration isolation (Figure 12a). In the isolation system with a Stewart cube, the actuators are perpendicular or parallel to each other. As shown in the figure, each support leg consists of an acceleration sensor and an actuator. Payload, which has a cylindrical shell structure and is considered rigid, is placed on the support legs. The base is designed as a combination of I-profile to improve the stiffness of the structure. The dynamic model of the system was constructed by using the Newton-Euler method (Figure 12b). The control strategy for this system is the LQR control method.^67^

**Figure 13:**
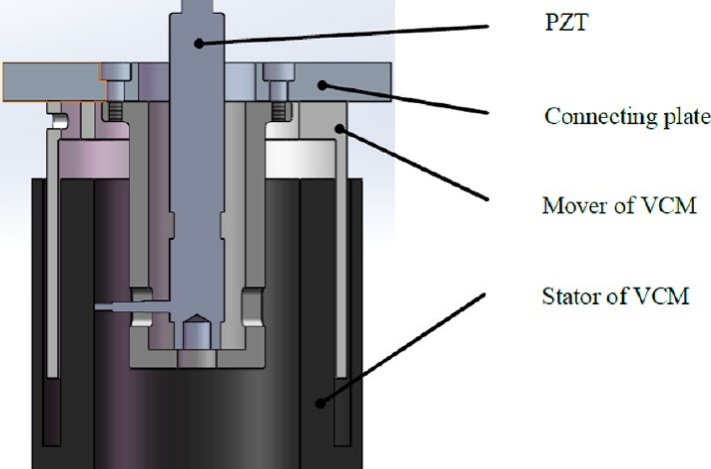
Proposed active vibration isolation system (DSA-AVIS). Reprinted with permission from ref (68). Copyright 2019 IEEE.

According to the proposed dynamic model, the function of the active vibration isolation system based on the piezoelectric actuator is given in eq 7 and the possible control force that the system will produce is given in eq 8.
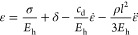
7Here, ε, *E*_h_, δ, *c*_d_, ρ, and *l* give the axial strain, modulus of elasticity, strain due to input voltage, stress, material damping, and length, respectively.
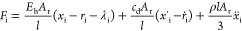
8Here, *F*_i_, *A*_r_, *x*_i_, *r*_i_, and λ_i_ represent the control force, cross-sectional area of the PZT, output displacement, base displacement, and output displacement generated by the voltage, respectively. Zhao et al. (2017), developed a dual stage active vibration isolation system, combination of a voice coil motor and piezoelectric actuator.^68^ The simulation and experimental results have shown that the proposed si*x*-axis orthogonal active vibration isolation platform with piezoelectric actuator can effectively reduce the dynamic response of an average of 5 dB payload in the frequency range of 20 Hz to 200 Hz. The active vibration isolation system presented in Figure 13, which has long stroke, high sensitivity, and wide band gaps.

**Figure 14:**
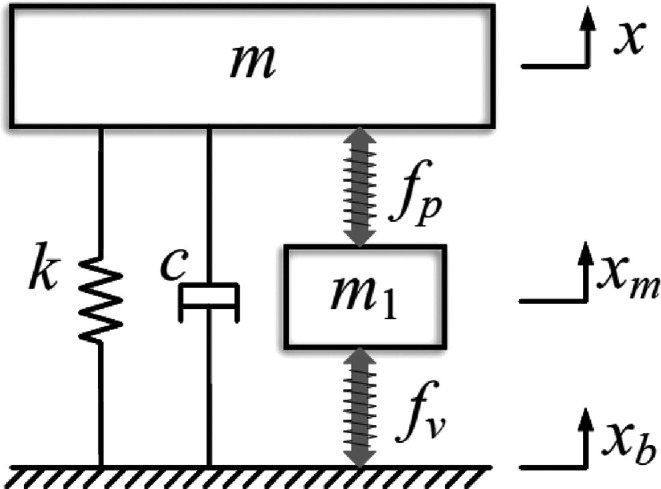
Dynamic model of DVA-AVIS. Reprinted with permission from ref (68). Copyright 2019 IEEE.

**Figure 15:**
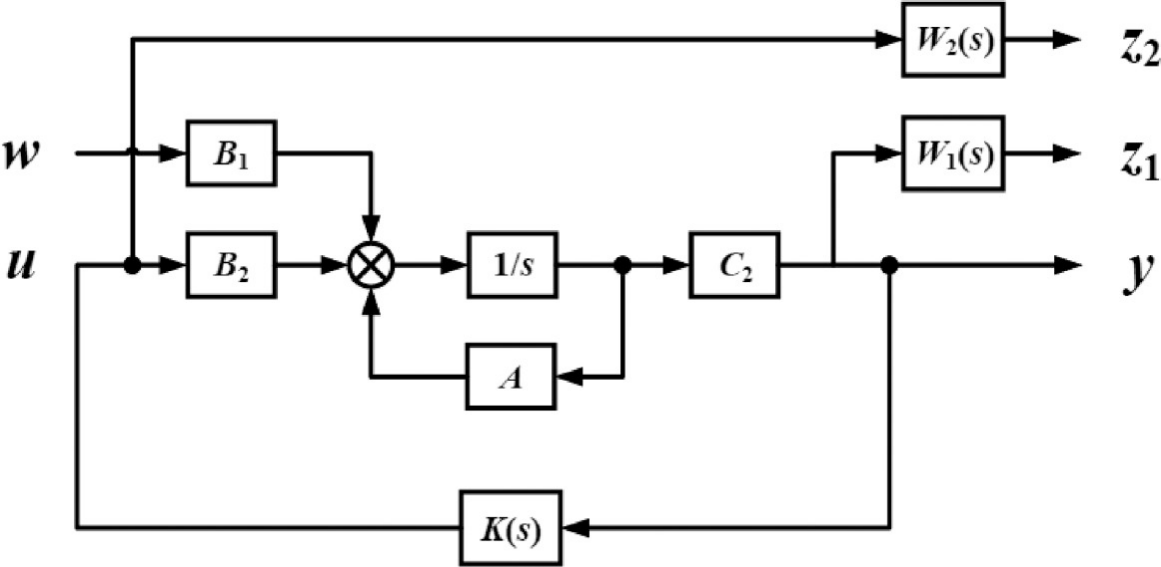
Control block diagram. Reprinted with permission from ref (68). Copyright 2019 IEEE.

The dynamic modeling of DSA-AVIS is presented in Figure 14. Here, *m* is the mass of the load, *m*_1_ is the mass of the VCM (voice coil motor) mover, PZT, and the coupling device, *k* and c are the stiffness and damping coefficient of the passive vibration isolation system, *f*_v_ and *f*_p_, the forces of the VCM and PZT, respectively, and *x*, *x*_b_, and *x*_m_ represent the displacement of the load, base, and mover, respectively. The dynamic general equations of DSA-AVIS are expressed in eqs 9 and 10 as follows:
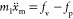
9
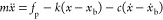
10The force calculated from VCM is calculated based on Faraday’s Laws as in eq 11.
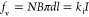
11Here *N* is the number of coil turns, *B* is the average magnetic induction of the air gap, *d* is the diameter of the coil, *I* is the current value of the coil, and *k*_i_ = *NB*π*d* is the repulsion coefficient of the VCM. The longitudinal voltage of the piezoelectric part is calculated as shown in eq 12.
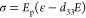
12Here *E*p is the modulus of elasticity, ε is the longitudinal strain, *d*_33_ is the longitudinal piezoelectric deformation coefficient, and *E* is the longitudinal electric field intensity. The operating voltage and longitudinal displacement of the PZT are given as
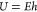
13
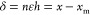
14where *h* is the thickness of the individual layers of a stack actuator and *n* is the number of stacked ceramic layers. The output power of the PZT was obtained as
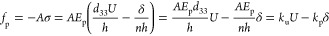
15The control block diagram of DSA-AVIS is given in Figure 15. Here *z*_1_ and *z*_2_ are control outputs. *W*_1_ and *W*_2_ reflect the troubleshooting performances and output range of the actuators, respectively. *T*_yw_ and *T*_uw_ show the transfer function from the disturbance input *w* to the measured output *y* and the transfer function from the disturbance input *w* to the control input *u*. Controller *K* is obtained by minimizing the *H*_∞_ norm of the transfer function from *w* to *z*_1_ and *z*_2_.
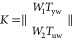
16DSA-AVIS consists of a spring, a payload, a PZT stack, a pair of bee-float rails, a tie plate, a VCM, and a base. The spring is used to balance the weight of the load. Air float rails can minimize friction in the vertical direction. PZT stacks and VCM are connected in series with each other. In addition, the disk spring used in DSA-AVIS is fixed between the PZT stack and the VCM in order to adjust the stiffness of the PZT. In the experimental setup in Figure 15, the input voltage is applied to the surface of the piezoelectric ceramics, causing the PZTs to lengthen or shorten. Thus, the force produced by the piezoelectric ceramics was applied with the help of the output velocity.

**Figure 16:**
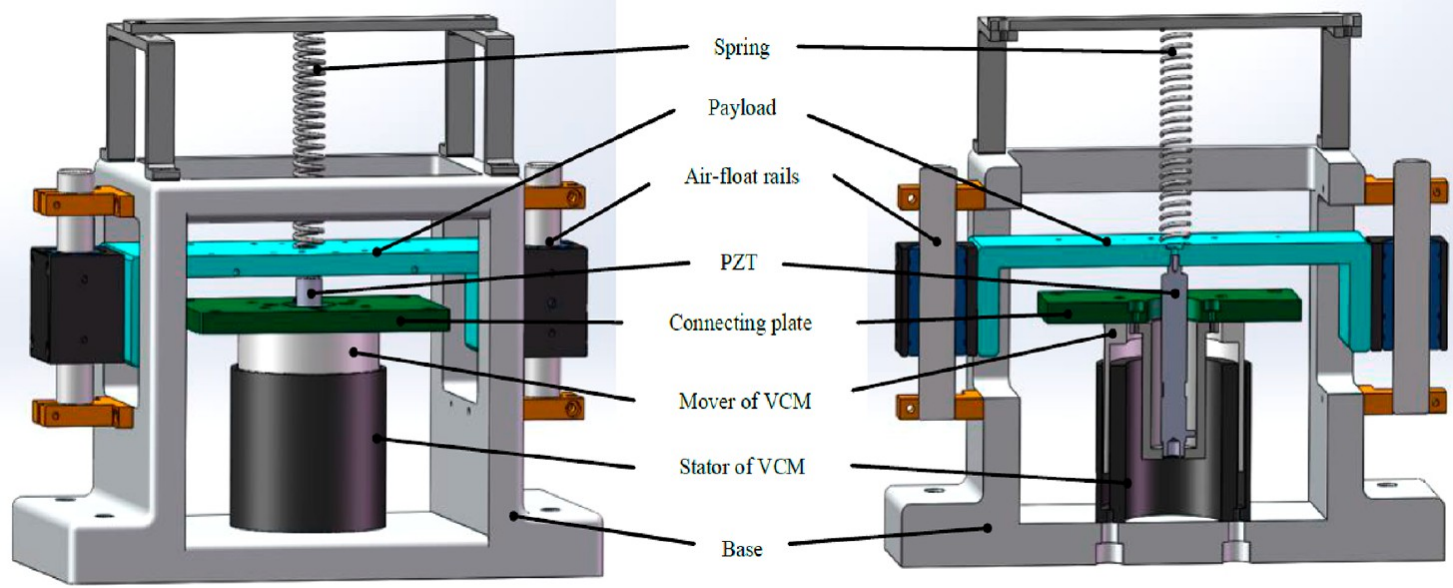
Experimental setups for DSA-AVIS. Reprinted with permission from ref (68). Copyright 2019 IEEE.

In Figure 16, the real-time target machine is the core component of the entire experimental system designed to send control instructions to the drivers of the VCM and PZT, respectively. TA115 type current amplifier and E01 type voltage amplifier act as drivers of VCM and PZT respectively. It also receives real-time target machine measurements from the GS-11D velocity sensor. The velocity sensor is fixed to the bottom of the experimental setup with the help of a magnet. The controller is based on the MATLAB/Simulink software. The simulation results showed that the closed-loop transmittance is less than −18 dB from 0 to 100 Hz. This means that the proposed *H*_∞_ controller DSA-AVIS designed has a good vibration isolation performance.

Wang et al. (2021) developed an innovative piezoceramic friction damper (PCFD) to provide a reliable energy dissipation device for structural response control. In PCFD, high-strength bolts limit the deformation of the piezoelectric ceramics under voltage and change the preload of the high-strength bolts with the reaction force. Thus, real-time friction adjustment is realized by adjusting the positive compression on the friction surface. In order to evaluate the energy dissipation capacity of PCFD at varying voltages, a hysteretic performance test was performed and the experimental results were compared with the theoretical values. The finite-element model of the PCFD was established to analyze the stress distribution of each component of the PCFD under different voltages. Based on the semiactive control features of PCFD, the Takagi-Sugeno fuzzy neural network semiactive control system is designed for frame structure with PCFD. The learning function of the adaptive network-based fuzzy inference system (ANFIS) is used to create fuzzy rules and fuzzy neural network controller (FNNC). Finally, semiactive control simulation of the three-story frame structure model under seismic stimulation was performed by Simulink. Experimental and numerical results showed that the PCFD designed in this study has good energy dissipation capacity, and the maximum control force increases linearly with increasing voltage. It has been found that the combination of FNNC and PCFD established by ANFIS can effectively reduce the structure response.^69^ Choo et al. (2022) designed a system using the vibration of a tuned mass damper (TMD) for use in bridge structures. The combination of TMD with piezoelectric technology is designed to create an ideal machine that can generate free and effective electrical energy in addition to the role of the passive control device.^70^

Within the light of the literature, the friction-based vibration isolation system is designed with piezoelectric actuators. With the piezoelectric friction dampers’ usage with control algorithms, such as PD/PID and LQG, the slit active vibration isolation system has emerged. For example, the control forces are generated against seismic vibrations from a coil system, excited by piezoelectric actuators. Against seismic vibrations, it is shown that the piezoelectric actuator takes an external power source and activates the friction forces. In addition, as a result of the use of tuned mass dampers (TMD), which is a passive damper, and piezoelectric actuators together, it has turned into a semiactive/hybrid control system. In the future, it is foreseen that semiactive or hybrid base isolation systems will be designed, and numerical and experimental studies will be carried out as a result of passive isolation systems benefiting from the energy efficiency of piezoelectric materials. However, in such a system, the selection of the right control algorithm to be used in order to prevent energy losses is very important. In addition, the optimization of piezoelectric actuators is necessary to obtain the energy to activate the semiactive or hybrid control system. Optimization analysis includes the type, length, thickness, width, and polarization of the piezoelectric material to be used as an actuator.

### 3.2 Structural Health Monitoring Applications

By monitoring the structural health of existing steel or reinforced concrete structures in earthquake zones, the evaluation of damage levels and structural integrity is possible with effective, easy-to-apply, and low-cost monitoring systems.^71^ It is used as a sensor in PVDFs that can be integrated into the wireless system with its flexibility, thin size, low-cost, and wireless system to monitor structures at a large scale in structural health monitoring systems.^72^ Lightweight layered piezoelectric transducers (PWA) have been developed to integrate PZTs used as actuators and sensors into the structure.^73,74^ Then, the light-layer-piezoelectric transducers’ sensors were developed.^75^ It is possible with this type of sensor to observe especially the crack type damages nondestructively with the Lamb waves.^76−78^ Alem et al. (2016) proposed a damage identification method in plate structures using embedded piezoelectric transducers and the Lamb waves. The damage index was determined to detect the presence and location of cracks.^79^ Apart from the electromechanical impedance, the PZT sensors are used in dynamic and wave propagation analysis.^80^ With the signals obtained by these methods, damage determinations are made with dynamic parameters; such as damping, frequency, and mode shapes of the structure. It is also necessary to consider the installation and cost of structural health monitoring studies made by combining sensors formed with piezoelectric materials and EMI techniques. To solve this problem, the so-called artificial sensor has been developed for smart aggregate structural health monitoring studies embedded in concrete.^74,81,82^ Song et al. (2011) designed concrete piezoelectric smart modules (CPSM) that resemble smart aggregates.^83^ CPSMs consist of waterproof RTV silicone and piezoelectric patches.^82,84,85^ CPSMs are made of piezoceramic materials. Thus, the voltage of the piezoelectric element is perpendicular to the surface with the best conductivity.^86^ The piezoceramic materials are preferred as an actuator or sensor, as a result of its high electromechanical and piezoelectric constant.^87^ The detection method using one of the CPSMs as a sensor and the other as an actuator is suitable for crack detection for concrete structures, as it is sensitive to cracks in the paths of voltage wave propagation.^88^ The piezoceramic actuator records the signal from the sensor. When a warning signal is observed, the signal change implies the damage (especially the crack) in the structure.^89^ Therefore, structural health monitoring and damage detection processes involve both direct piezoelectric and reverse piezoelectric effects.^90^ The relationship between the piezoelectric material and the structure is achieved either by affixing the piezoelectric material to the surface of the structural member with epoxy or by embedding it into the structural member. Piezoelectric materials adhered to the surface are likely to lose their functionality when exposed to a temperature or humidity. In this case, it is considered more reasonable to embed the piezoelectric material inside the structural member.^91^

There are many studies on the piezoceramic materials in structural health monitoring systems. Voutetaki et al. (2012) monitored concrete members reinforced with FRP under dynamic load using smart piezoelectric materials.^92^ Xu et al. (2013) monitored the structural behavior of concrete-filled steel elements using PZT sensors.^93^ Hughi et al. (2015) investigated the effect of crack width on monitoring systems through reinforced concrete tube and reinforced concrete beam experiments.^94^

**Figure 17:**
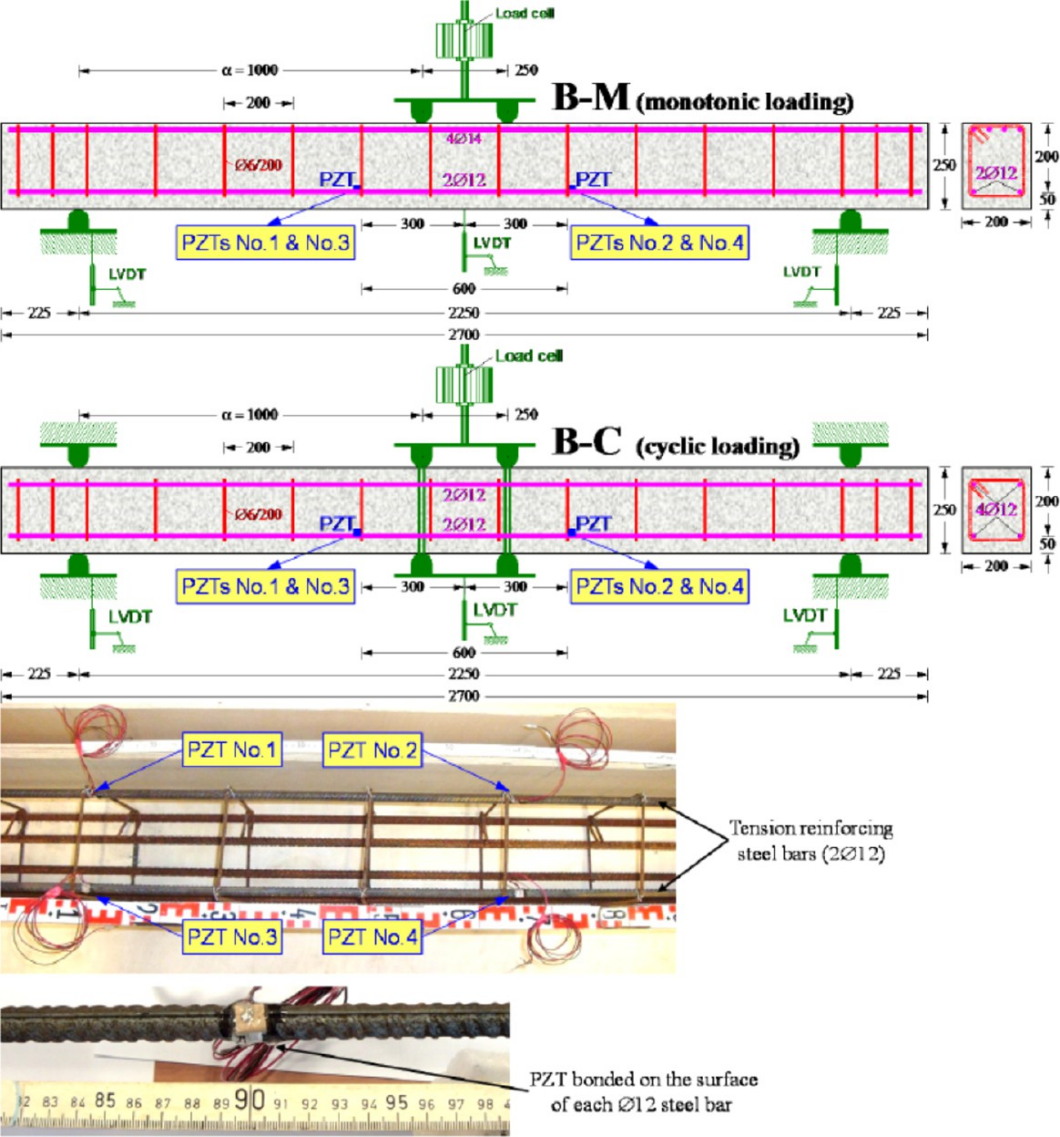
Test setup reinforced concrete beam. Reprinted with permission from ref (95). Copyright 2015 WIT Press.

Chalioris et al. (2015) experimentally applied a wireless earthquake damage monitoring system (WiAMS) by using PZT transducers in reinforced concrete beams.^95^ The damage detection by piezoelectric transducers (actuators/sensors) is based on the electromechanical impedance method using analytical integration. The piezoelectric lead zirconate titanate (PZT) type transducers were attached to the reinforcement surface of two large-scale beams (Figure 17). The damages were monitored according to the well-known root mean square deviation (RMSD) method, before and after yielding at different loading levels for the tested beam.^96^ RMSD defined as
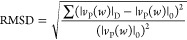
17Where |*v*_P_(*w*)|_D_ is the voltage value of the peak of the piezoelectric material. |*v*_P_(*w*)|_0_ is the base value of the absolute value of the peak voltage of the voltage signal (healthy state).

**Figure 18:**
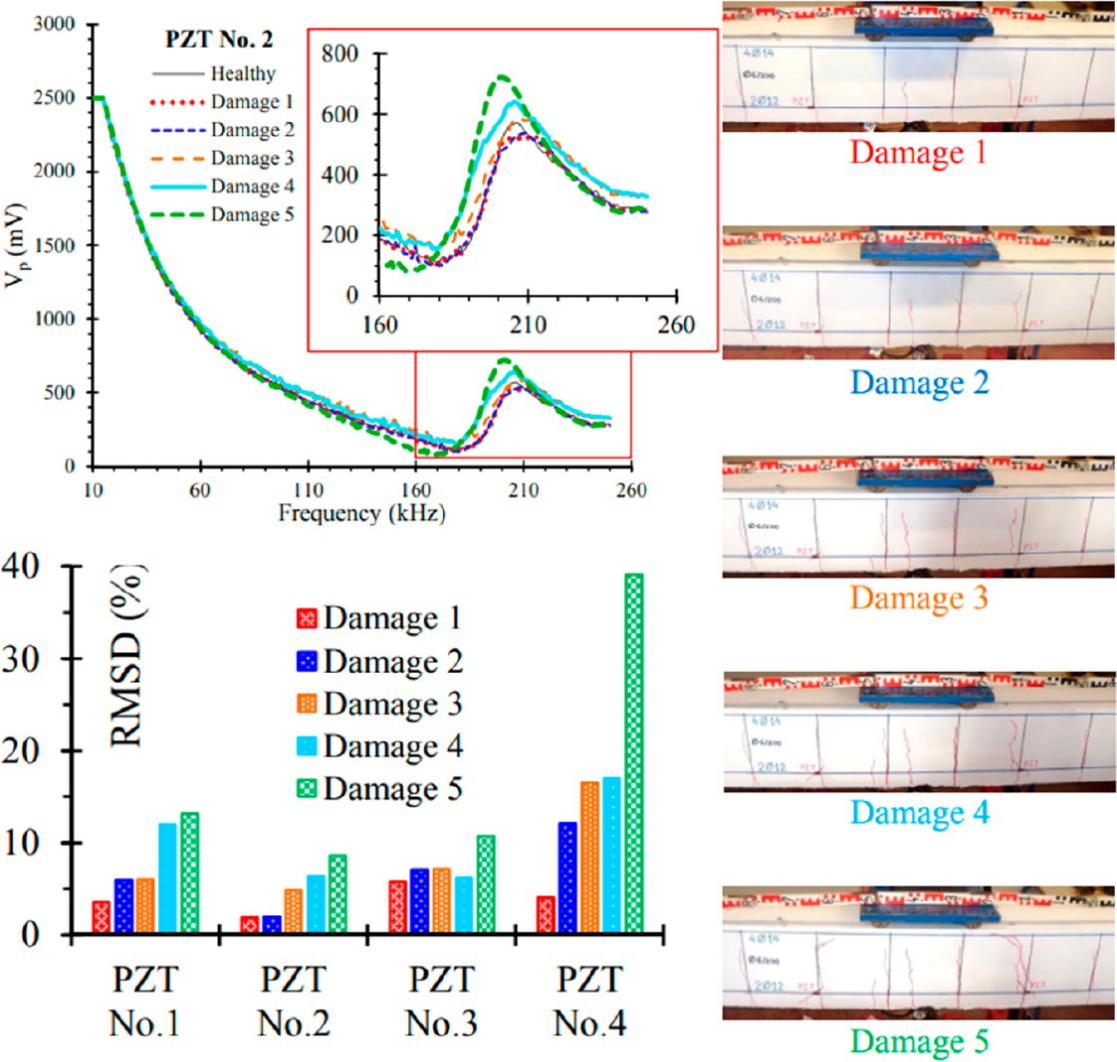
Test results of reinforced concrete beams. Reprinted with permission from ref (95). Copyright 2015 WIT Press.

The results showed that the use of PZTs to detect earthquake damage in reinforced concrete structures using the electromechanical impedance approach can be considered as a highly promising nondestructive structural health monitoring method. In Figure 18, voltage signals read in PTZs, as a result of monotonic loading applied to reinforced concrete beams, are shown. It is shown that the voltage values of the damaged and undamaged beams are different from each other. The results of the study showed that damage detection can be done with voltage drops.

Zhang et al. (2016) monitored concrete cracks in highways and airlines with a piezoelectric active sensing system.^97^ Jothi et al. (2016) conducted a structural health monitoring study with the EMI technique on reinforced concrete shear walls connected to PZT. Cahill et al. (2018) used aluminum cantilevers and an energy harvesting console designed from PVDF material to monitor the conditions of a bridge structure. According to the test results of the permanent magnet shaker, one of the energy collectors, voltage outputs of 0.0631 and 0.182 V, were obtained at natural frequencies of 6.2 and 20.6 Hz, respectively.^98^

**Figure 19:**
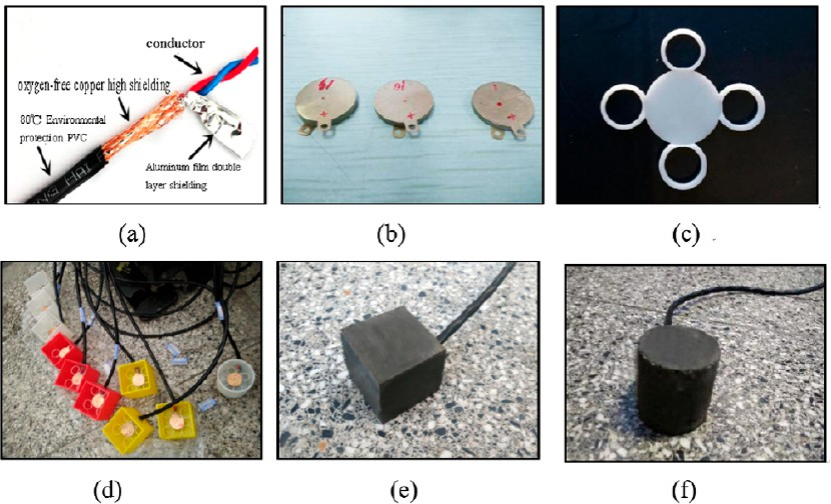
Production stages of the piezoelectric sensor: (a) conductive wire, (b) piezoceramics, (c) the producing molding of piezoceramics, (d) the coupling with wire, (e) the cube sample of piezoceramic, (f) the cylinder sample of piezoceramic. Reprinted with permission from ref (99). Copyright 2017 Elsevier.

**Figure 20:**
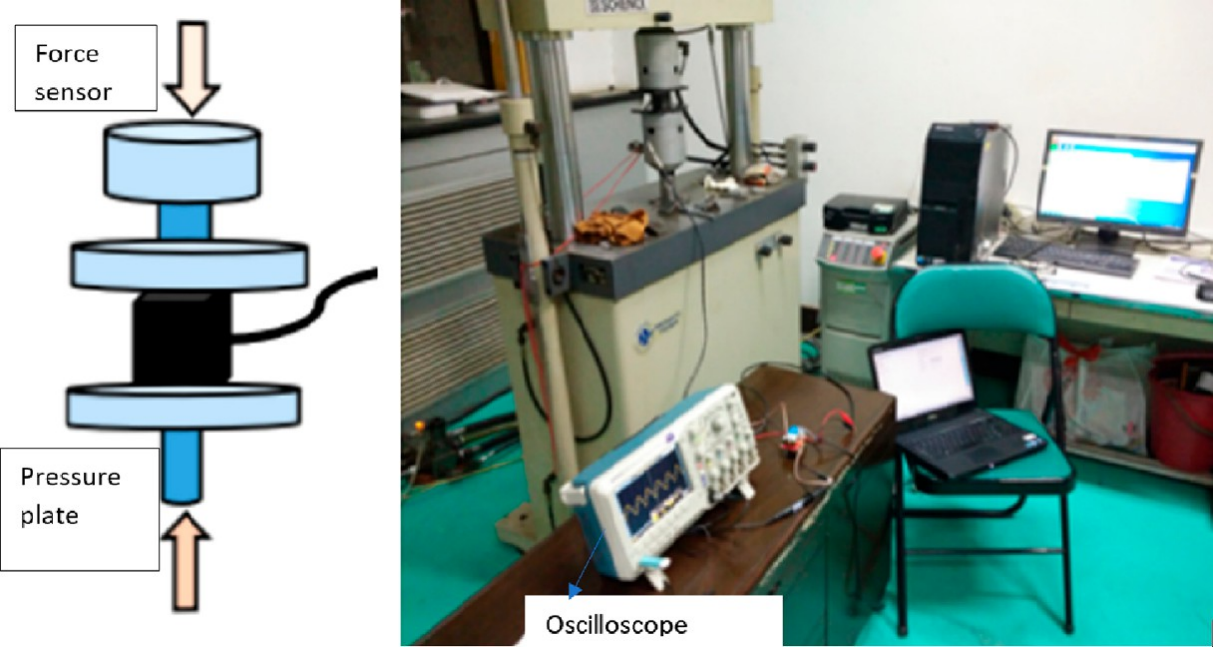
Piezoelectric sensor test setup. Reprinted with permission from ref (99). Copyright 2017 Elsevier.

Song et al. (2016) have been developed a new aggregate-shaped embedded piezoelectric sensor to examine the structural health for infrastructures (Figure 19).^99^ The sensor is composed of two phases, the functional phase and the packaging phase. The functional phase is designed by using a piezoelectric ceramic chip, and the packaging phase is designed using a new composite material. The frequency independence, linearity, sensitivity, response velocity, and service performance of the sensor were tested by frequency sweep and amplitude sweep (Figure 20).

Experimental results showed that within the vibration frequency range of common civil engineering structures the new aggregate-shaped embedded piezoelectric sensor has good mechanical and machinability properties. In addition, the new embedded sensors have been found to have very good mechanical-electrical coupling performance, which provides a solid foundation for further applications in civil engineering infrastructures.

**Figure 21:**
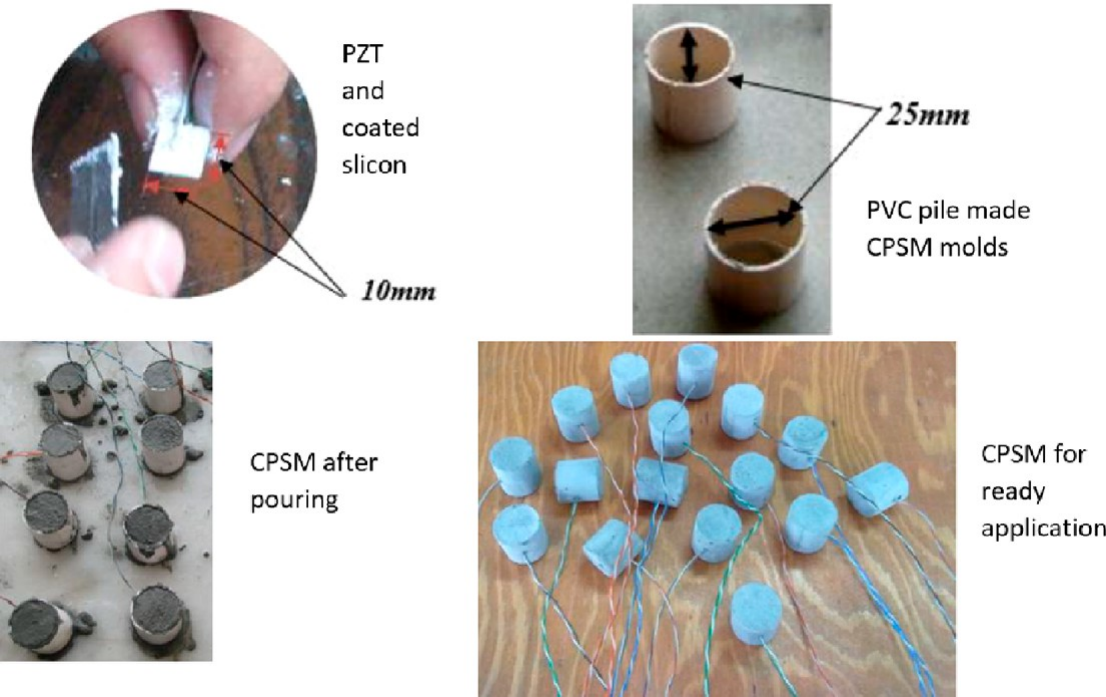
Production stages for CPSMs. Reprinted with permission from ref (100). Copyright 2017 Korea Science.

**Figure 22:**
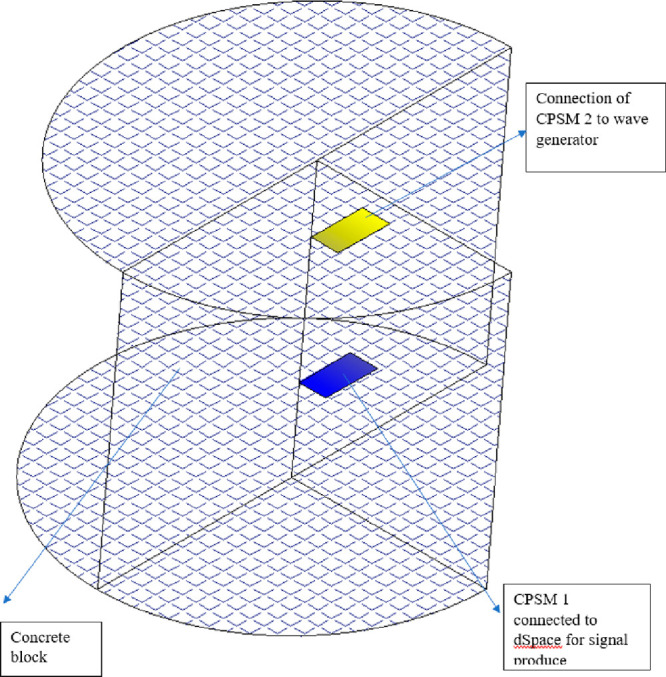
Concrete block and placement of CPSMs.

Zhang and Su (2017) conducted three main experiments to evaluate the applicability of engineered artificial piezoelectric transducers, called concrete piezoelectric intelligent module (CPSM) (Figure 21), in the dynamic structural analysis.^100^ Experimental modal analysis (EMA), based on the Abraham time domain (ITD) method, was applied for the extraction of model parameters, by using the finite-element type numerical analysis.^101^ First, CPSM was used to obtain the signals emitted in the concrete produced from the wave generator with a certain frequency and to compare the frequencies of the original excited signal. The layout of the CPSMs is presented in Figure 22. One side (upper) is connected to the wave generator to emit a signal, and the other side (lower) is connected to the dSPACE system for signal reception.

Evaluation was made by comparing the frequencies between the evoked linear chirp signal and the signal obtained by CPSM. Fast Fourier Transform (FFT) is applied to transfer the signal from time domain to frequency domain. The power spectral density (PSD) of both the chirp signal and that obtained by the CPSM are compared. The results showed that the higher intensity frequencies were in the same range. The PSD for the received signal for CPSM has been found to have a lower value compared with the chirp signal due to complex noise.

**Figure 23:**
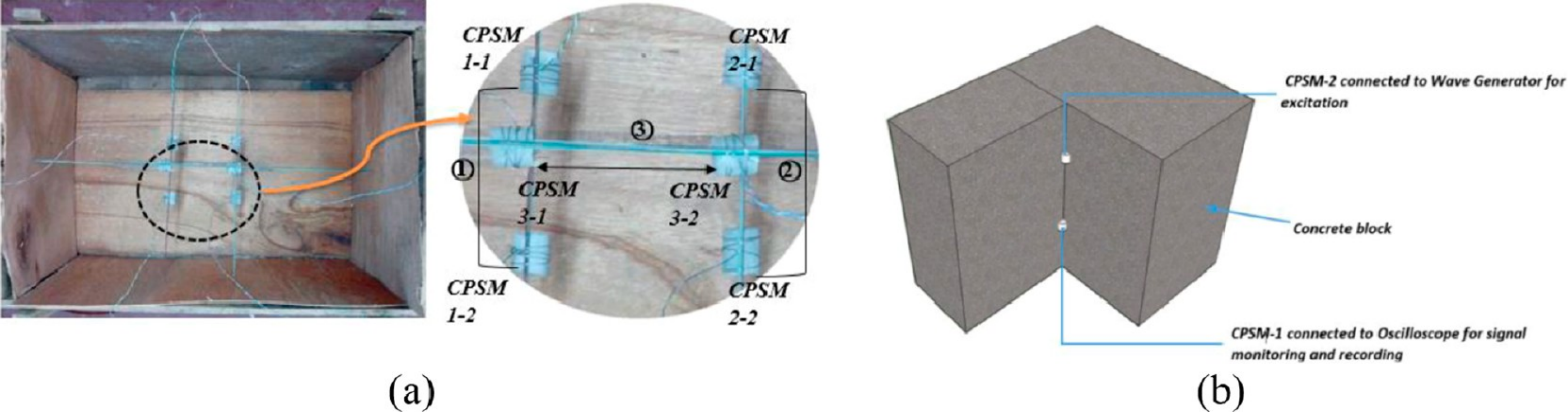
Experiment setup for the qualitative determination of the strength of concrete: (a) the placement mold of CPSMs, (b) monitoring the strength of concrete during hydration. Reprinted with permission from ref (100). Copyright 2017 Korea Science.

As the second experiment, a large concrete block was poured in order to qualitatively determine the strength during the hydration process (Figure 23). A wave generator is connected to the other CPSM to monitor the resulting waves (signals) and record the corresponding signal amplitudes, to generate signals that will stimulate the CPSM to emit pressure waves into the concrete.

The obtained signal amplitudes were recorded by monitoring them for 12 different time points during the concrete hydration period. Depending on the amplitude, angular frequency and wave density of the signal obtained from the CPSMs, the concrete strength was determined using the following equations.^101,102^
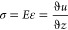
18
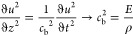
19
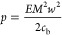
20
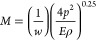
21In the equations, *M* is the amplitude of the harmonic wave, w is the angular frequency, *p* is the power of the harmonic wave, *E* is the Young’s modulus of the concrete, and ε is the unit shortening.

**Figure 24:**
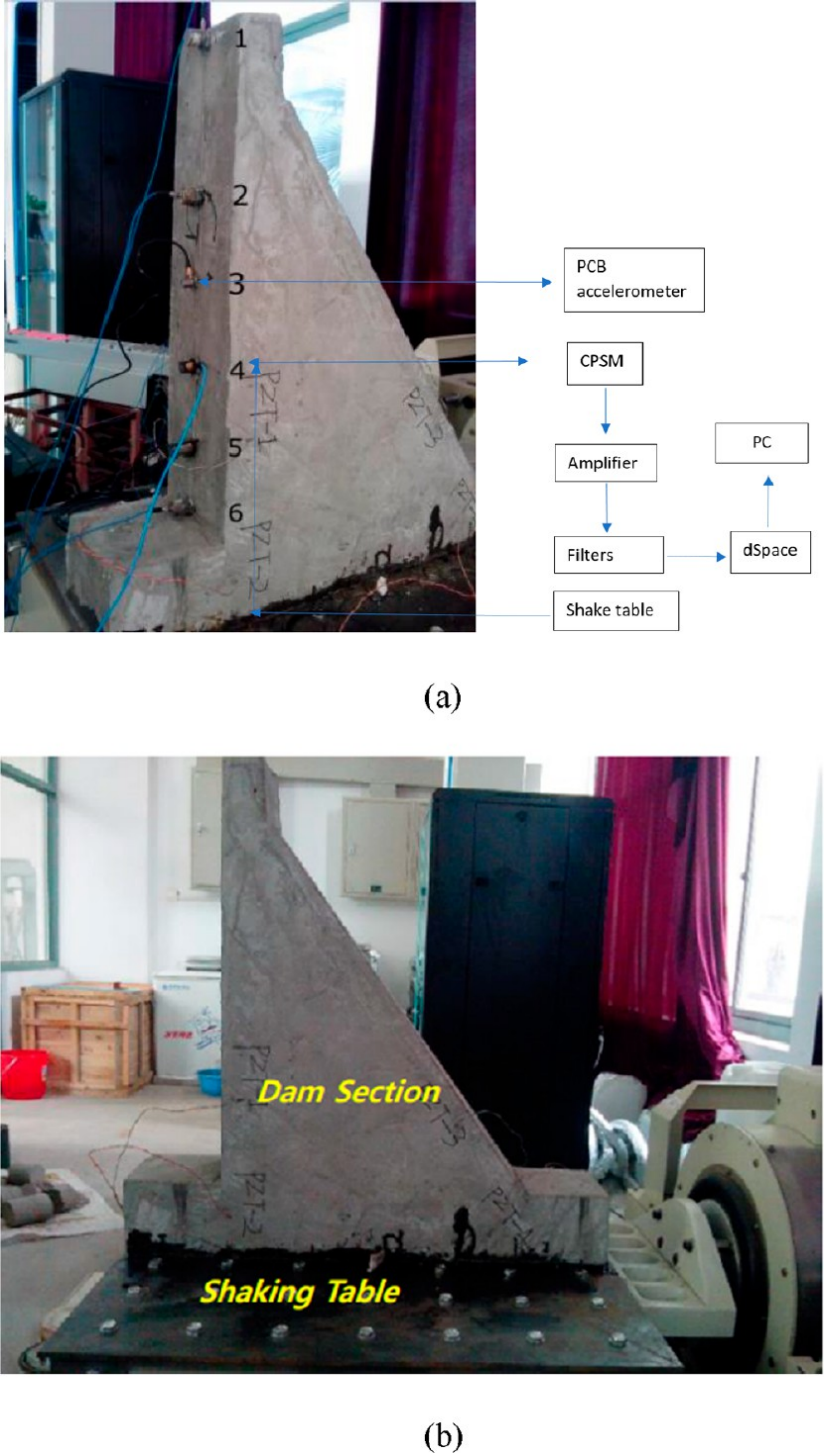
Experimental setup for the monitoring system: (a) the placement of the accelerometer of the dam section, (b) the application of the shake table. Reprinted with permission from ref (100). Copyright 2017 Korea Science.

In another study, structural health monitoring was carried out by means of PCB sensors placed on the dam module surface formed with CPSMs. The aim is to detect if the signals obtained from the CPSM are similar to the signals obtained from the PCB sensors. The structural health monitoring of the experimental setup given in Figure 24 was carried out with the help of a shaking table. Experimental modal analysis in the study is based on the Abraham time domain technique (ITD) introduced by İbrahim and Mikulcik (1973).^103^ In addition, the finite element model of the dam module was established, and the numerical and experimental results were compared by making a modal analysis.

Modal analysis and experimental modal analysis results showed that the modal natural frequencies extracted from the signal obtained from CPSM overlapped with the modal natural frequencies extracted from the signal obtained from PCBs.

**Figure 25:**
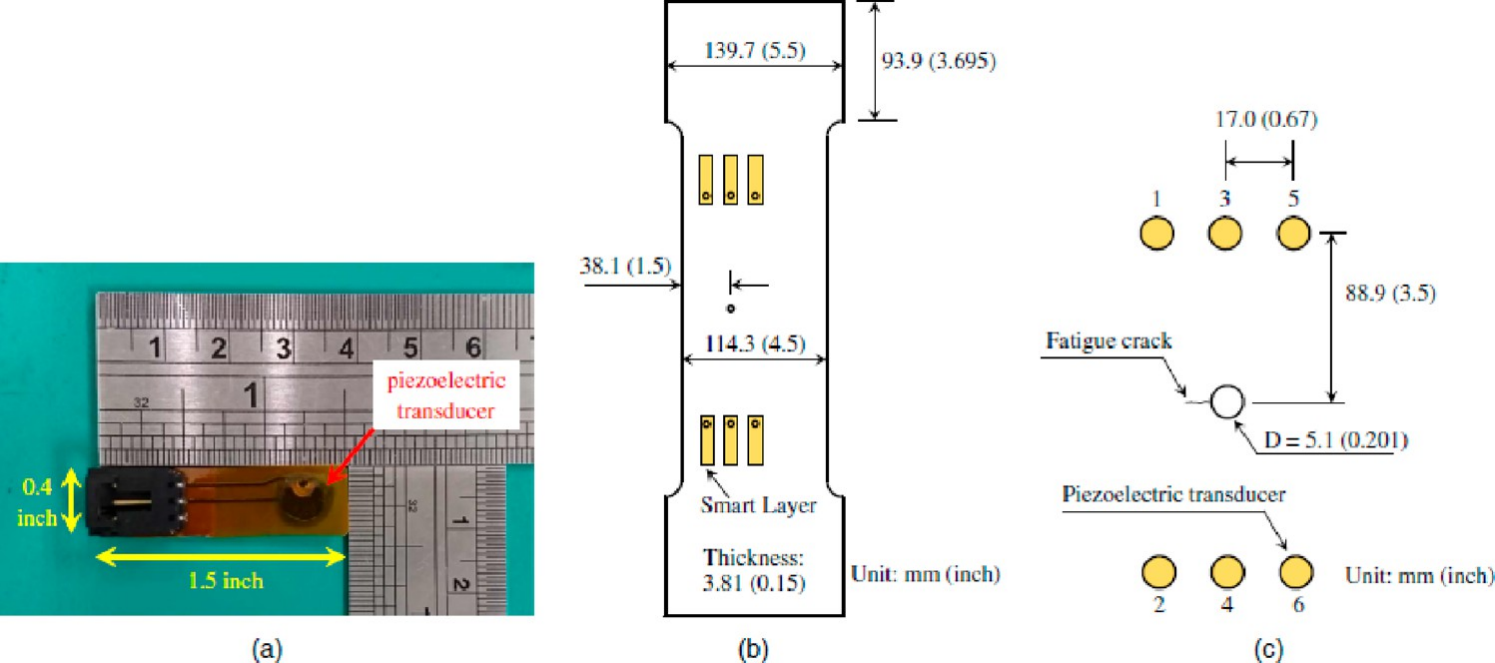
(a) Smart layers in experiments; (b) elements of the metal part; (c) detailed location and dimensions of piezoelectric transducers. Reprinted with permission from ref (104). Copyright 2021 American Society of Civil Engineers.

**Figure 26:**
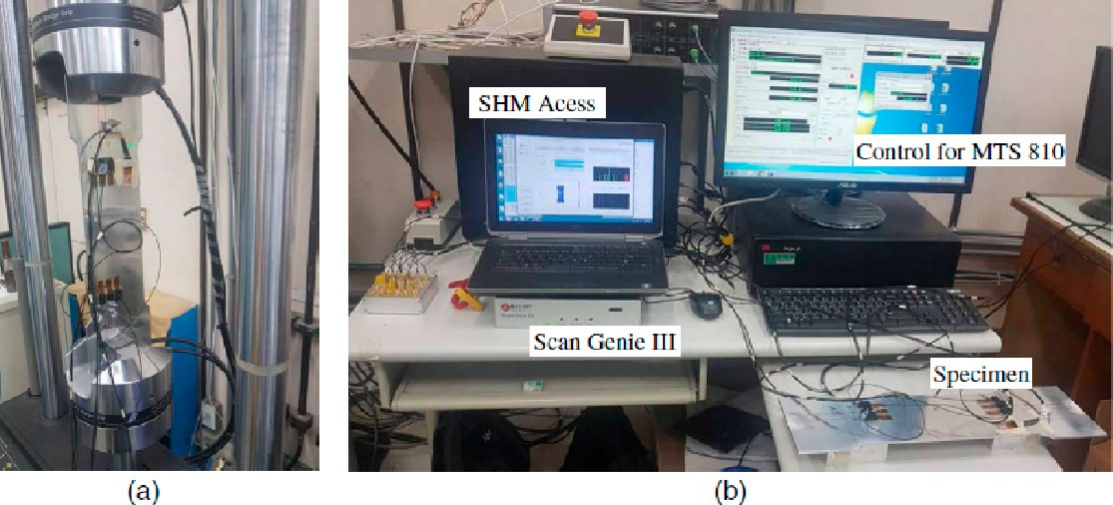
Experimental setup for metallic crack monitoring: (a) experimental setup, (b) structural health monitoring mechanism. Reprinted with permission from ref (104). Copyright 2021 American Society of Civil Engineers.

Chen et al. (2020) investigated fatigue cracks in metallic structures with lamb waves produced by the use of piezoelectric transducers.^104^ Tests were performed on three samples to determine the fatigue crack and damage indices. In each sample, a transducer array consisted of six Stanford Multiple Actuator-Receiver Transmission (SMART) Layers. All transducers were piezoelectric and mounted near the fatigue crack (Figure 25). Averaging has been proposed to determine the damage index of the piezoelectric transducer. Lamb waves with different frequencies were tested to perform damage monitoring (Figure 26). A universal testing machine (MTS 810) was used to demonstrate the fatigue crack propagation. A moving microscope (Dino-Lite pro) was used to monitor the actual crack length during the fatigue test, also. In addition, SHM Access version 2.17 software and ScanGenie III hardware were used to actuate and detect Lamb waves and calculate damage indices.

The experimental results showed that the plot of the mean damage index against the actual crack length reveals a narrow band that indicates a small standard deviation of the measurement data. Lamb wave with *F* = 450 kHz driving frequency was found to perform best in crack tracking.

**Figure 27:**
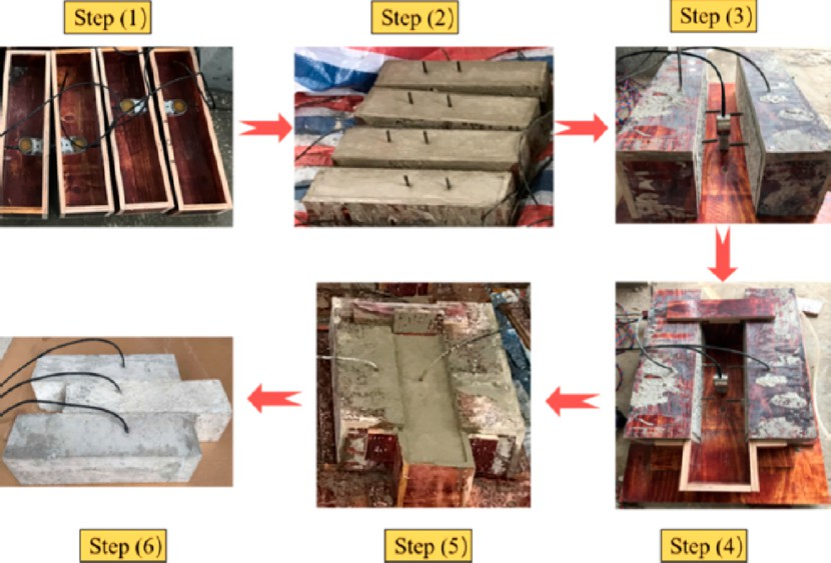
Production phase of structural members with piezoelectric-based smart aggregates. Reprinted with permission from ref (105). Copyright 2021 American Society of Civil Engineers.

**Figure 28:**
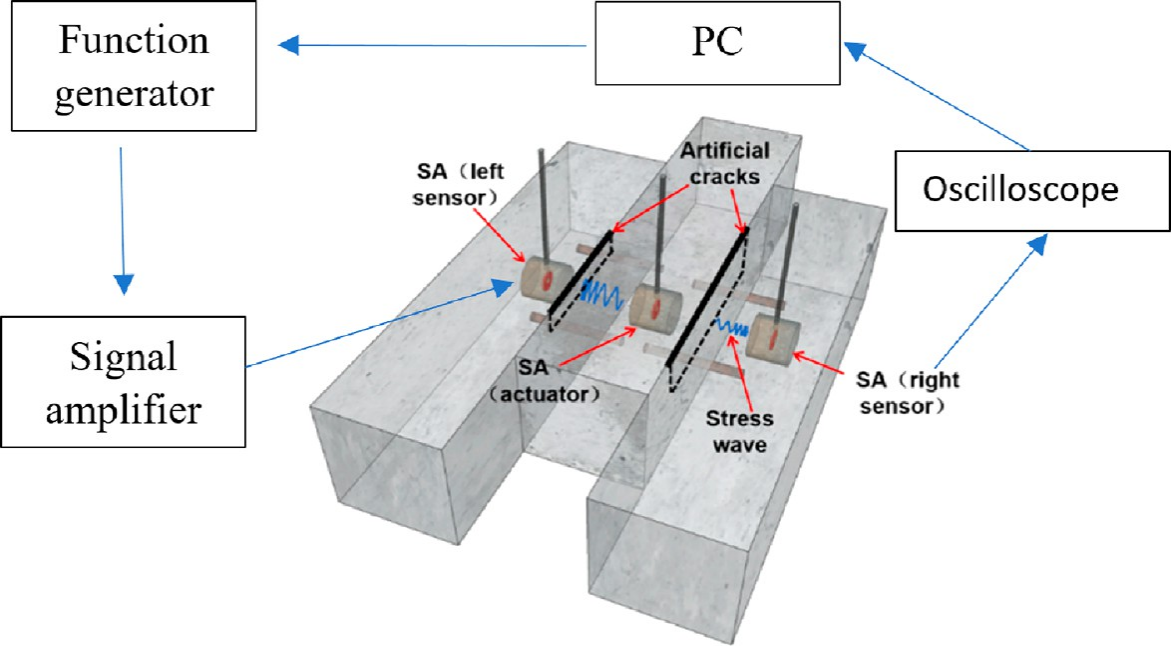
Flowchart of the monitoring system with piezoelectric based SA. Reprinted with permission from ref (105). Copyright 2021 American Society of Civil Engineers.

Jiang et al. (2021) worked on the damage observation through the interface of the laminated concrete, manufactured by using piezoelectric smart aggregates.^105^ The push-out experiments were realized at the interface of laminated matter by using an active sensing perspective. The piezoelectric smart aggregates are presented at Figure 27. These piezoelectric smart aggregates are checked by sensors and/or actuators. Then, the signals within a selected frequency range are processed specifically by transferring them from function generators to signal amplifier. The predicted stress signals were applied to structural members to observe the damage level by predicting the signal energy reduction ratio, the damage index. The experimental setup is given at Figure 28. The results showed that the proposed damage index can effectively detect the presence and severity of cracks in the laminated concrete interface. It is shown that the proposed approach is applicable to monitor the occurrence and development of crack damage and to estimate the crack size at the layered interface.

Deng et al. (2021) applied a piezoelectric ceramic (PZT) based electromechanical impedance (EMI) method to detect the damage at the CFRP-concrete interface. In the study, the separation process of the RC beam reinforced with a CFRP plate under four-point loading was monitored by EMI method.^106^ Pan et al. (2022) proposed a nondestructive testing method using piezoelectric sensors to monitor the stress–strain relationship of concrete using the electromechanical impedance technique. Two piezoelectric sensors (a lead zirconate titanate (PZT) sensor and a piezoelectric cement (PEC) sensor) were investigated. Compression tests were performed to obtain a 28-day stress–strain curve by using a high-resistance strain gauge. In addition, the impedance–frequency spectrum of the piezoelectric sensor embedded in the concrete was measured with an increment of 0.1 fc at each load. The results showed that as the load increased, the concrete conductivity measured by the PEC and PZT sensors at the applicable frequency decreased. Therefore, it is concluded that both sensors can be used to monitor the voltage during loading.^107^

It is desired that structural health monitoring studies should be effective, easy to implement, and low cost. In addition, it is not desired to use a technique that can change any dynamic parameter (mode shapes, frequency, etc.) of the structure and cause damage to the structure in building health monitoring studies. Therefore, the use of nondestructive (NDI) tests such as Lamb waves and electromechanical impedance (EMI) has become widespread in recent years. As a result of combining the properties of piezoelectric materials such as displacement, deformation, and stress sensing capabilities, low cost, high conversion efficiency, and lightness with these techniques, local damage detection studies were carried out in structural members. By using piezoelectric materials as sensors, monitoring of cracks in reinforced concrete beams, concrete, concrete-laminated interfaces, and metallic structures has been carried out. In addition, the early wet strength values and stress–strain curves of the concrete material were obtained as a result of the use of piezoelectric materials as both as sensors and embedded aggregates. In many studies, it has been observed that the signals detected by normal piezoelectric sensors overlap very closely with the signals received from embedded piezoelectric aggregates. It will be important to detect some local damage in advance in structures that may be exposed to destructive vibrations, such as earthquakes. Therefore, using piezoelectric materials as sensors to detect damages in multistory structures is expected to be done in future studies. In addition, it is thought that the use of embedded piezoelectric aggregates in the elements of structures to be built in earthquake zones may be a precursor for early damage detection. In short, detecting structural damage with piezoelectric materials and applying appropriate strengthening/repair processes will make the structures more protected against earthquakes.

### 3.3 Energy Harvesting Applications

Energy harvesting systems studied over the years have been one of the best techniques to solve the global energy problem without consuming natural resources.^108^ An energy harvesting system has two main elements, a microgenerator that converts ambient energy into electrical energy, and a voltage amplifier that pumps it by regulating the generator voltage.^109^ Piezoelectric materials, generally used to generate electrical energy, come into our mix as PZT and PVDFs.^110^ The mechanical energy transformation to the electrical energy, conveys the piezoelectric materials as transducers in energy harvesting systems.^50,111,112^ The longitudinal piezoelectric coefficient *d*_33_ is high in PZT and PVDF, preferred in power generation applications.^113,114^ However, since piezoelectric materials are suitable for low frequency conditions in energy harvesting systems, they are not sufficient to power devices operating at high frequency.^115^ The modulus of elasticity of PZT is 25 times that of PVDF and has a higher resonance frequency than PVDF while having the same geometry.^116^ However, the fragility of PZT is thought to be an alternative way to use PVDF in energy harvesting systems.^117,118^ Although, the PVDF does not have as high-voltage conversion coefficients as PZT, being a flexible material is seen as an advantage in energy harvesting systems.^119^ Researchers have used piezoelectric materials in energy harvesting systems.^120^ Zhao et al. (2011) analyzed piezoelectric stacks and electrical energy transfer units under a cymbal force in a finite-element environment, and obtained 1.20 MW power at 20 Hz frequency at 0.7 MPa stress.^121^ Kumar et al. (2013) designed an energy harvesting ground module with a piezoelectric material with a high coupling coefficient. They found that connecting piezoelectric materials in parallel and increasing the number of piezoelectric modules per unit area would provide higher voltage output.^122^ Ghosh et al. (2016) eliminated the polarization problem of piezoelectric materials in high-frequency environments by designing a wireless self-powered ferroelectric nanogenerator (FTNG) and a porous polymer composite membrane-based nanogenerator (PPCNG).^123^ Saxena et al. (2017) analyzed the effect of mass shape on power generation performance of the cantilever piezoelectric transducers and designed a pyramidal mass structure.^124^

Many researchers in the world literature have conducted many studies on energy harvesting systems obtained by using piezoelectric materials on roads.^125^ Generally, the piezoelectric energy collection units are used to collect vibration energy from vehicles, people, or other objects on the roads.^126^ The collection of kinetic energies originating from human walking is one of the methods of providing electricity to low-power devices by applying energy harvesting technologies that have the potential to be a power generator in the individual.^127^ Yoshiyasu (2008) developed an energy harvesting device embedded in the pavement.^128^ He explained that when the traffic volume is 500, he obtains up to 250 kW of electrical energy per km. Xiong et al. (2012) showed that the deformations caused by the processing of vehicles and vehicle movements on pavements can be used in the generation of electrical energy.^129^ Najini et al. (2013) conducted theoretical studies on the feasibility of existing piezoelectric transducers for piezoelectric power coatings by optimizing suitable piezoceramic types for road mechanical energy harvesting. Yuan et al. (2014) tested piezoelectric power converters in railways and obtained 30 MW of power.^130^ Wang et al. (2015) prepared two types of piezoelectric power producing asphalt concrete consisting of piezoelectric and asphalt (pavements) materials to realize energy harvesting from pavements. The results showed that the piezoceramic material with 35 mm diameter and 0.4 mm thickness can meet the technical requirements of power coating. They obtained 14 V as an electrical output response.^131^ Yang et al. (2017) analyzed the most compatible piezoelectric transducer in a finite-element environment to obtain energy from road traffic.^132^ Izrin et al. (2017) designed a power converter using a half-wave rectifier for raindrop energy application to convert the damping alternating present (AC) produced by PVDF to direct current (DC). Basically, raindrop energy was generated by converting the kinetic energy of the raindrop into electrical energy-using polyvinylidene fluoride (PVDF) piezoelectricity.^133^

**Figure 29:**
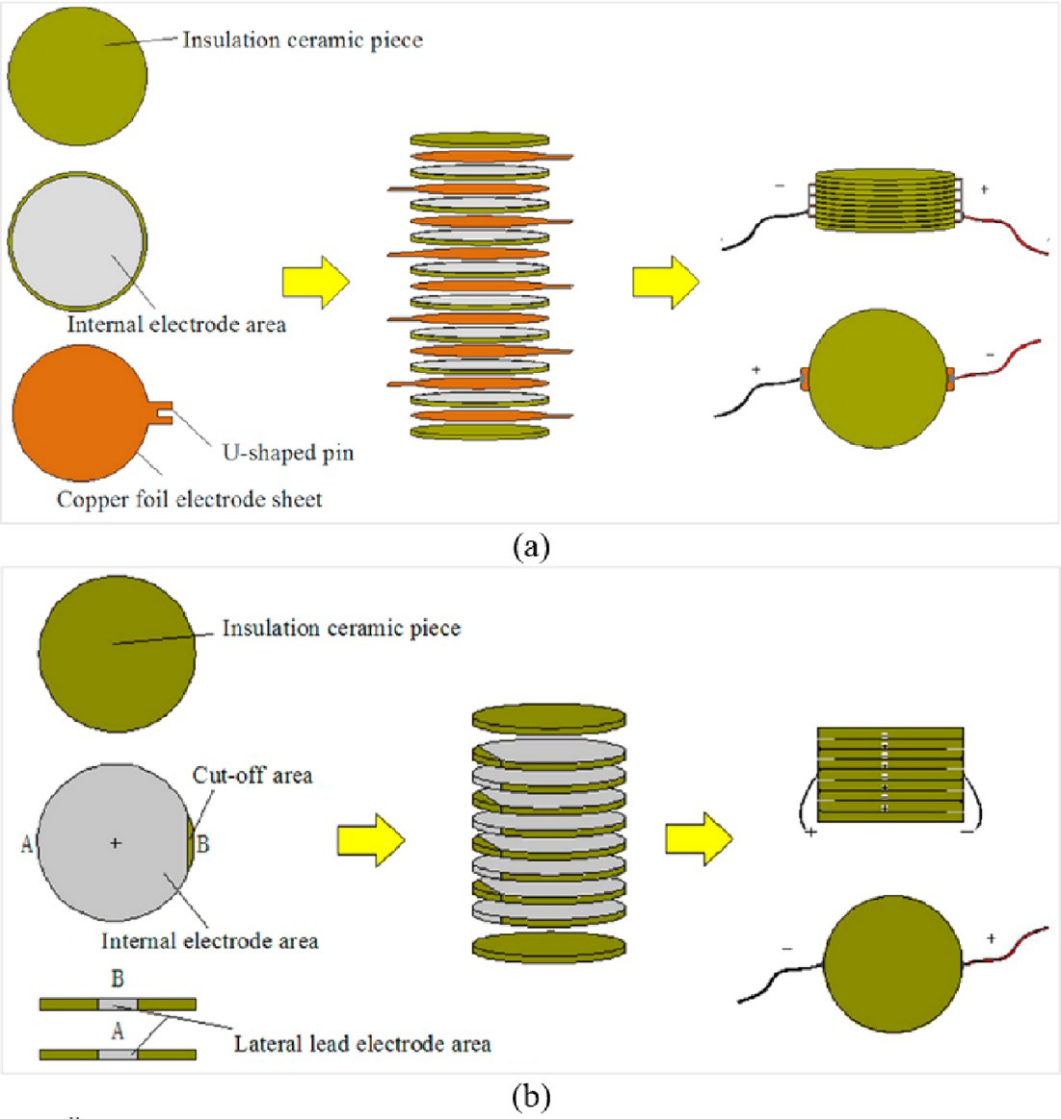
Proposed piezoelectric energy harvesting structures: (a) U-shaped interlayer copper foils, (b) lead-containing lateral electrode structure. Reprinted with permission from ref (134). Copyright 2019 Elsevier.

Wang et al. (2018) proposed two types of U-shaped interlayer copper foil electrode and lateral lead electrode structure. Their aim was to examine the performance of units designed with piezoelectric stacks for energy harvesting from pavements.^134^ A U-shaped interlayer copper foil electrode is shown in Figure 29a. It is proposed to reduce the short-circuit effect and increase the quality, mass production, and efficiency of wire welding in layers, efficiently. A copper foil was placed between each piece of the piezoelectric ceramic and electrically connected to the upper and lower silver electrodes of the piezoelectric ceramic piece by means of a conductive silver paint glue. Each copper foil has a U-shaped pin at one end, which acts as an external electrode of the interlayer copper foil to weld the wire. The interlayer copper foil electrode structure is suitable for piezoelectric stack with relatively few piezoelectric ceramic layers. If more layers are to be stacked, the corresponding number of copper foil electrodes will reduce the effective thickness of the entire piezoelectric stack and weaken its connection with the path structure. Therefore, a lateral electrode, as presented in Figure 29b, is also proposed. The silver electrode area of each piezoelectric ceramic is extended to the side end surface, and the wires are directly welded to the side end electrode area.

**Figure 30:**
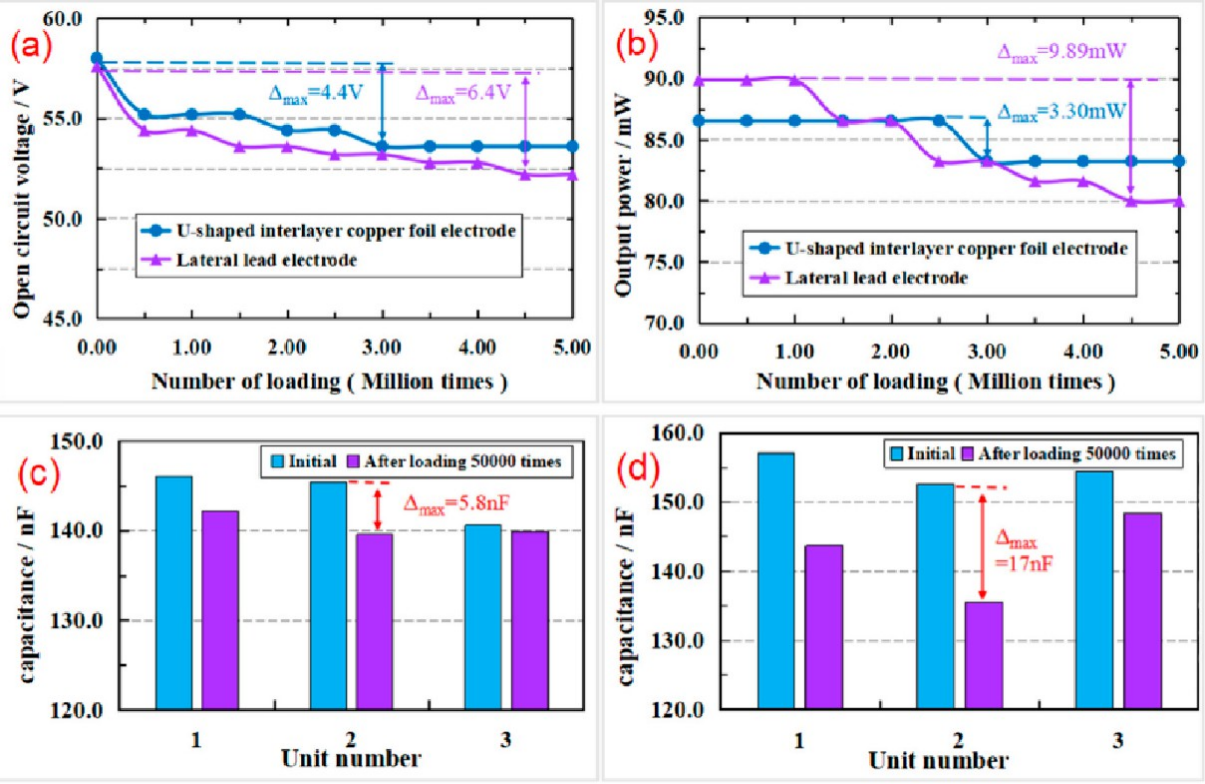
Test results of energy harvesting systems. Reprinted with permission from ref (134). Copyright 2019 Elsevier.

Results have been determined that the optimal load resistance of the units with U-shaped interlayer copper foil electrode and lateral lead electrode under 0.7 MPa stress reaches approximately 20 kΩ. The mechanical test results after 50 000 simulations (MTS) cyclic loading showed that the structural performance of the U-shaped interlayer copper foil units is relatively durable and stable. Test results are given in Figure 30.

**Figure 31:**
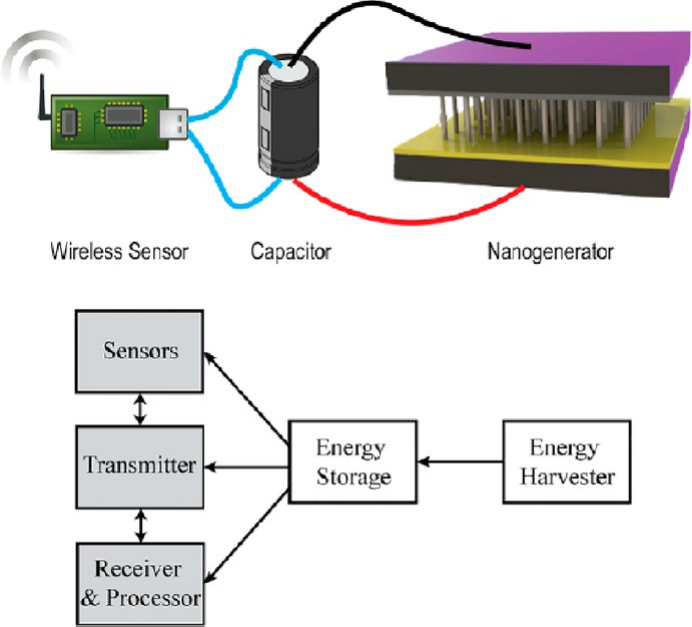
Energy harvesting from piezoelectric generators and self-powered sensors. Reprinted with permission from ref (7). Copyright 2019 Elsevier.

Apart from energy production on roads, there are energy production applications in buildings. Piezoelectric materials can be used in renewable and sustainable structures that can produce their own energy and sense themselves thanks to their ability to convert mechanical tension and vibration energy into electrical energy. Therefore, buildings subject to severe vibration and huge dynamic loads from wind, earthquake, traffic, and human activities are considered the most suitable candidates for applying piezoelectric energy harvesters and sensors.^135,136^ Energy harvesting is the theorem that enables unused and wasted energy resources to be converted into electrical energy and used in relevant places.^137^ Piezoelectric energy harvesting systems are examined in three groups among themselves as macro- and medium scale, micro electromechanical systems (MEMS) and nano scale. As shown in Figure 30, macro and medium harvesting systems often power small electrical devices such as wireless network transmitters and sensor nodes. MEMS scale harvests can power submicrometer-sized chips and structural components. Nanometer-scale harvesters harvest energy via synthesized nanowires and can be fabricated as self-powered sensors, as shown in Figure 31.^138,139^

**Figure 32:**
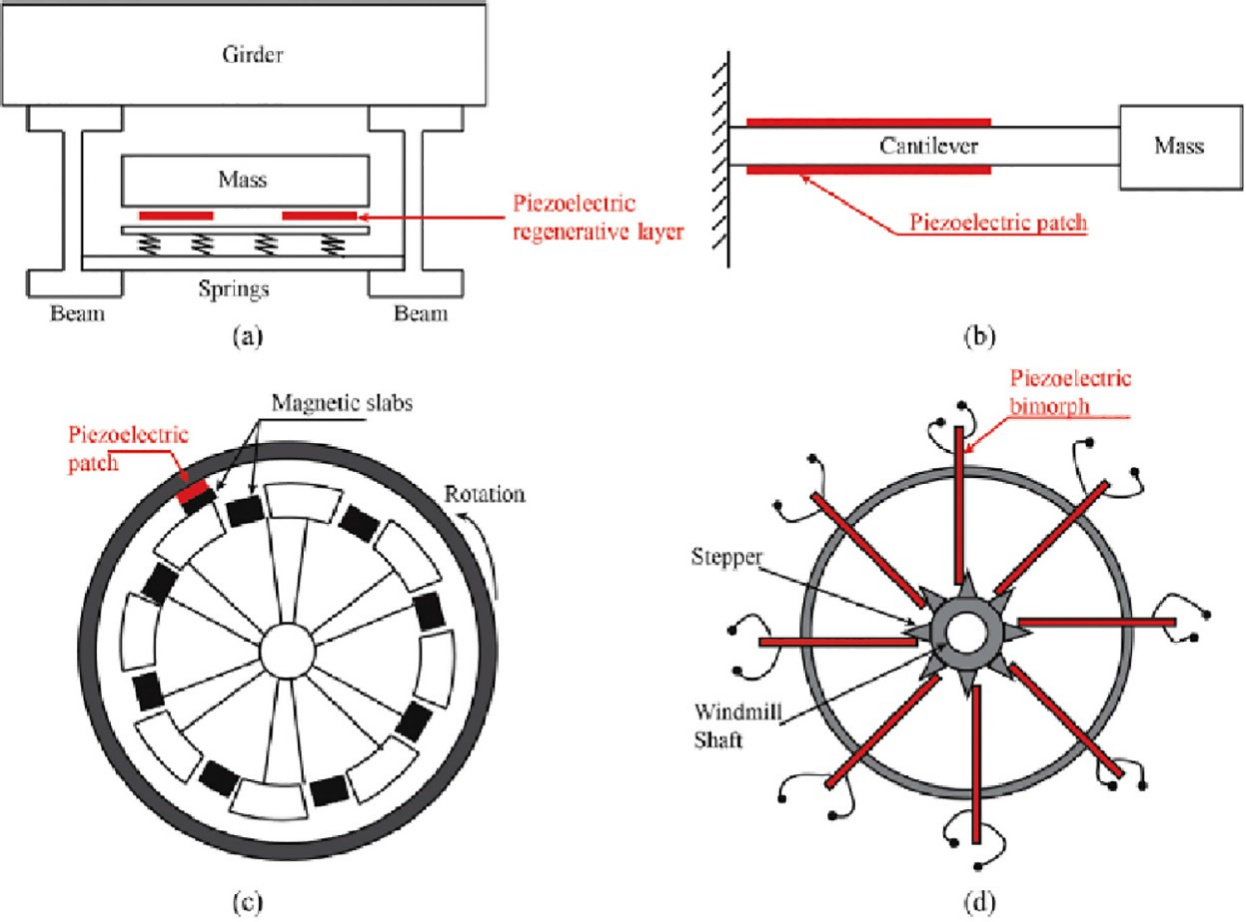
Piezoelectric structural elements: (a) piezoelectric damper, (b) piezoelectric cantilever, (c) piezoelectric harvester, (d) piezoelectric windmill. Reprinted with permission from ref (7). Copyright 2019 Elsevier.

In recent years, researchers have proposed combining vibration control with energy harvesting.^140^ For example, piezoelectric coupled cantilever structures have been used not only to harvest energy but also to help dissipate the vibration of buildings as tuned mass dampers, as shown in Figure 32a.^141^ Xi et al. (2015) examined these structures under harmonic loading and proposed a generator design by connecting two piezoelectric patches in series as shown in Figure 32b.^142^ Piezoelectric harvesters can also be arranged in a ring that is compatible with high excitation frequencies and suitable for varying radius sizes, as shown in Figure 32c. Xi et al. (2014) obtained a total of 5274.8 W power from buildings in their research.^143^ To absorb vibration energy from wind and use in energy harvesting system, piezoelectric devices have more advantages than wind turbines due to their compactness, robustness and simple installation.^144^ Priya et al. (2005) developing a piezoelectric windmill technology based on bimorphic actuators as shown in Figure 32d, they achieved 7.5 mW of power from 10 mph wind speed.^144,145^

**Figure 33:**
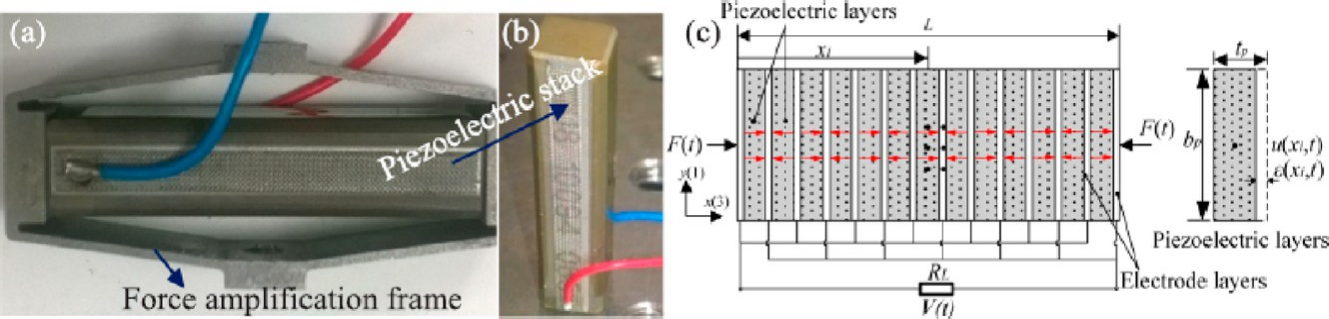
(a) Piezoelectric stack transducer with force amplification frame, (b) piezoelectric stack, (c) electromechanical coupling model. Reprinted with permission from ref (146). Copyright 2018 Elsevier.

Qian et al. (2018) presented a distributed parameter model for a multilayer piezoelectric stack transducer based on the axial vibration theory of a continuous rod (Figure 33).^146^ In Figure 32, both the tension and compression frames are specifically designed to increase the power output from the piezoelectric stack means. A piezoelectric stack transducer with a pull frame is shown in Figure 33a. Figure 33b and c shows the shape of the piezoelectric stack and an equivalent electromechanical model, respectively. In the stack, the piezoelectric layers with the total p number are polarized along the x(3) direction and are overlapped in series with the electrode layers connected in parallel, as shown in Figure 33c.

**Figure 34:**
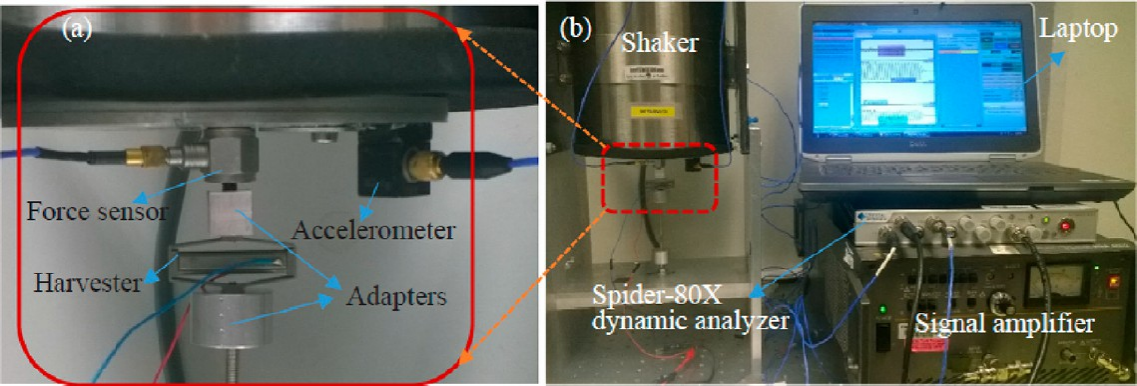
Experimental setup: (a) layout of the harvester, (b) general experimental setup. Reprinted with permission from ref (146). Copyright 2018 Elsevier.

Experiments and numerical simulation show that the presented analytical model is reliable in predicting the electrical responses of the piezoelectric bulk collector (Figure 34). It has also been found that the voltage and power generated at different external resistances increase as the random excitation level.

Energy harvesting is a system that can prevent energy production by consuming natural resources and without releasing too much CO_2_ that can cause global warming in the world. The studies examined have shown that piezoelectric materials are preferred in energy harvesting applications due to their small size and high power density, as well as obtaining electrical energy from mechanical stress and dynamic vibrations. Finite element models of the piezoelectric energy harvesting systems proposed in the studies were established, and some dynamic and static analyzes were applied. These systems are generally designed to harvest energy from dynamic vibrations such as wind, the energies of people walking, railways, and pavements. In order to maximize energy harvesting from piezoelectric materials, the longitudinal piezoelectric coefficient, *d*_33_, should be as high as possible. In order to strengthen this coefficient, it is thought that piezoelectric materials should be strengthened with conductive materials, such as graphene. It is also foreseen that piezoelectric materials operating at a low frequency should be strengthened with conductive materials in order to produce energy at a high frequency. It should not be forgotten that piezoelectric materials must have high strength and deformation ability at the same time in order to obtain energy by using piezoelectric materials from applications that require high-power density such as dynamic vibrations, mechanical stresses, and vehicle loads. In the future, it is predicted that by designing piezoelectric materials with conductivity, strength, and deformability, applications where energy can be produced in a higher-frequency environment can be made. In addition, it is thought that shape and size optimizations should be made in order to obtain high energy from piezoelectric materials. It is thought that the use of piezoelectric materials as sensors and actuators and at the same time as the basic material for energy harvesting will become widespread for the global warming caused by the consumption of natural resources and structural safety, which is also the main problem of civil engineering. In other words, the energy to be obtained from a piezoelectric energy harvesting system suggests that it will be used both to meet the energy requirements in buildings, and it can also be a power source for active or semiactive control systems.

## 4 Future Perspective of Piezoelectric Materials

It is expected that piezoelectric materials will be preferred as the most important smart material of the last century in order to reduce the consumption of natural resources in terms of producing energy that can be used by humans. It is predicted that the use of piezoelectric materials for energy harvesting from mechanical vibrations will become commonplace with no carbon dioxide emissions. The fact that the Earth is in a state of constant vibration supports this fact. As is known, natural disasters such as earthquakes cause intense destruction in structures and cause loss of life. System identification, structural health monitoring, and structural control strategies have been developed to protect both buildings and people from the damage caused by earthquakes. It is possible to monitor the cracks and strength losses in the structure as a result of using piezoelectric materials as microelectromechanical sensors. In addition, it is preferred in structural control applications in terms of producing control forces as a result of using it as an actuator under the influence of electrical energy. In the last century, it is thought that the use of piezoelectric materials as both actuators and sensors will be used more effectively in reducing the damage to the structure caused by earthquakes. It is also expected to provide electrical energy savings, with buildings to be designed from conductive structural elements that can generate electrical energy by themselves. It is estimated that necessary studies can be carried out for a structure to produce its own energy with piezoelectric materials and to activate the relevant control mechanism by monitoring the health status of the structures with this material technique. Piezoceramic PZT and polymer PVDF are the prominent among these materials. It is an important parameter that PZT produces high energy and plays a role in both structural health monitoring and structural controls in high frequency domains. On the other hand, since PZT is predicted to break at high dynamic vibrations, it is expected that PVDF, which is more flexible than PZT, will be used. However, due to the difficulty of polarization of PVDF, it was thought that different alternatives should be considered. It is expected that some studies will be carried out to ensure the polarization feature of PVDF in dimensions that will resist dynamic vibrations.

## 5 Conclusions

This review covers the applications of piezoelectric materials in civil engineering. First, the technical properties of piezoelectric materials were mentioned. These applications were evaluated as structural control strategies, base isolation systems, structural concrete applications, structural health monitoring, and energy harvesting systems. An extensive literature review is evaluated and compiled. As a result of the examinations, it has been determined that the piezoceramic lead zirconate titanate (PZT) material has a higher piezoelectric coefficient, voltage coefficient, and strain coefficient. However, PZT material has some disadvantages, such as its brittle structure and negative effects on the environment due to its lead content. Barium titanate and PVDF polymer materials were considered suitable as alternatives to the PZT material. Among these materials, PVDF stands out as the most outstanding material due to its flexibility and high-energy production efficiency. However, in PVDF, the polarization needs to be strengthened by supporting it with some conductive materials. In the study, then, the usability of piezoelectric materials in structural control strategies, structural health monitoring systems, and power generation systems was investigated. It has been determined that actuator or sensor designs are made by using PZT material in general. It has been understood that PZT is generally used in the design of actuators that can generate control forces to control structures under the influence of dynamic loads, such as earthquakes and winds. In addition, structural health monitoring studies were carried out with the help of PZT sensors attached to the surface of existing structures. Here, voltage outputs can be measured in PZT materials that detect signals applied to the structure by signal analyzers. It has been determined that damage determinations can be made with empirical relations by means of the measured voltage outputs. It has been determined that high-power outputs are also obtained as a result of using piezoelectric materials as sensors or generators in structures that are under the influence of any random and harmonic excitation. It has been observed that the most preferred piezoelectric materials in energy harvesting applications in buildings are PZTs. However, the reasons such as the fact that PVDF can be shaped more quickly, processed, and produced higher energy than PZT strengthens that PVDF can function as a sensor, actuator, and generator in structural and earthquake engineering. It is thought that many applications can be made in civil engineering with the use of PVDF in the future, since there are very few controls, structural health monitoring, and energy generation studies in the literature with the use of PVDF. However, in order for piezoelectric materials to be used in structure control systems operating in higher-frequency environment, it is very important to polarize them by size optimization.
